# Supplementary material for: Engineering biosynthesis of the anticancer alkaloid noscapine in yeast
Source: Nat Commun. 2016 Jul 5;7:12137. doi: 10.1038/ncomms12137 (PMC4935968; doi:10.1038/ncomms12137)
Supplement: Supplementary Information — Supplementary Figures 1-14, Supplementary Tables 1-8 and Supplementary References [file ncomms12137-s1.pdf]

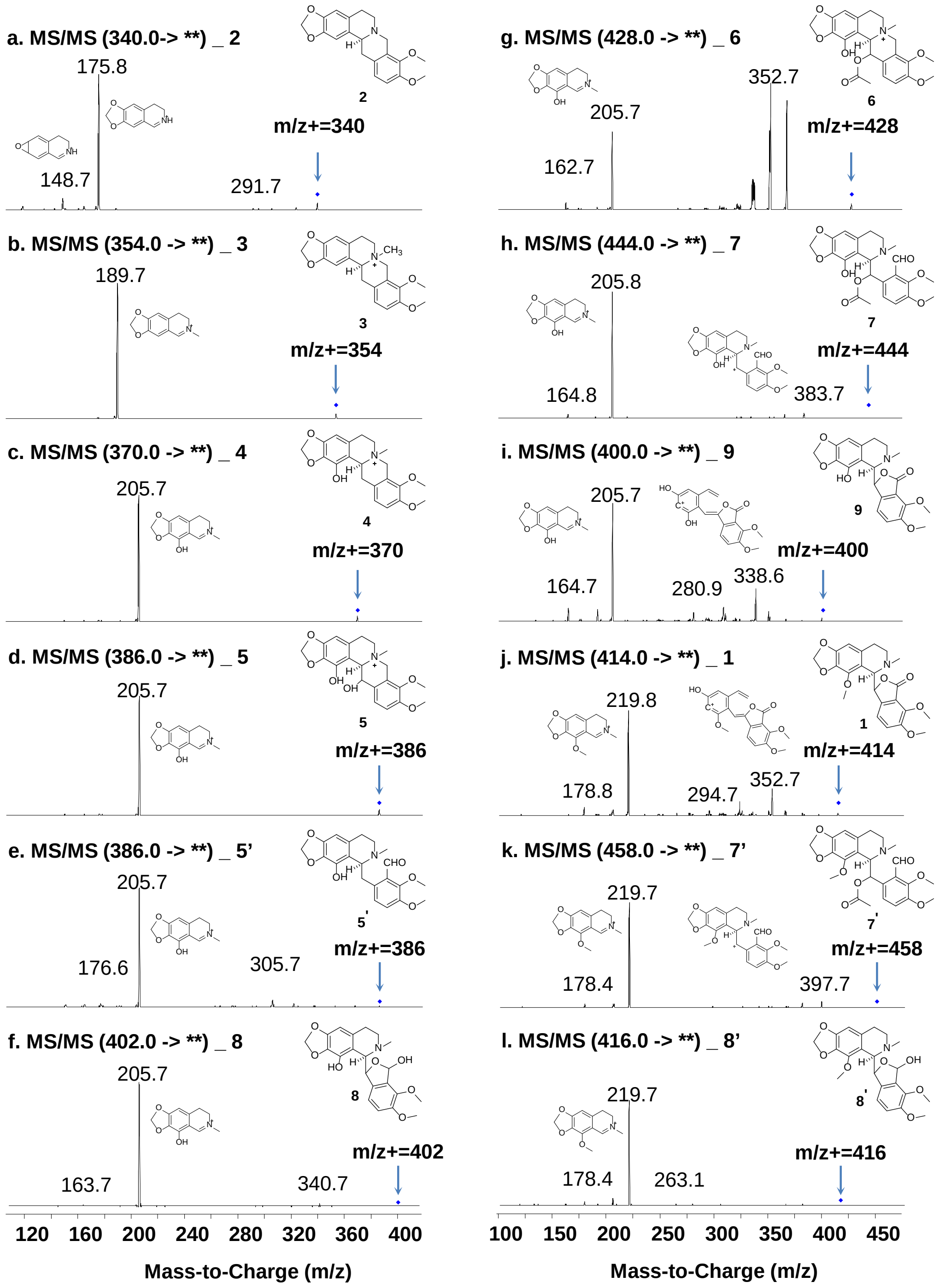

Supplementary Figure 1. MS/MS spectra of noscapine pathway intermediates

The MS/MS spectra of **(a)** canadine, **2**, **(b)** N-methylcanadine, **3**, **(c)** 1-hydroxy-N-methylcanadine, **4**, **(d)** 1,13-dihydroxy-N-methylcandine, **5**, **(e)** 4'-O-desmethylmacrantaldehyde, **5'**, **(f)** narcotolinehemiacetal, **8**, **(g)** 1-hydroxy-13-O-acetyl-N-methylcanadine, **6**, **(h)** 4'-O-desmethyl-3-O-acetylpapaveroxine**,** **7**, **(i)** narcotoline, **9**, **(j)** noscapine, **1**, **(k)** 3-O-acetylpapaveroxine**,** **7'**, **(l)** narcotinehemiacetal, **8'** were compared with those of standards or the reported spectra[1,2] to verify their identity.

a

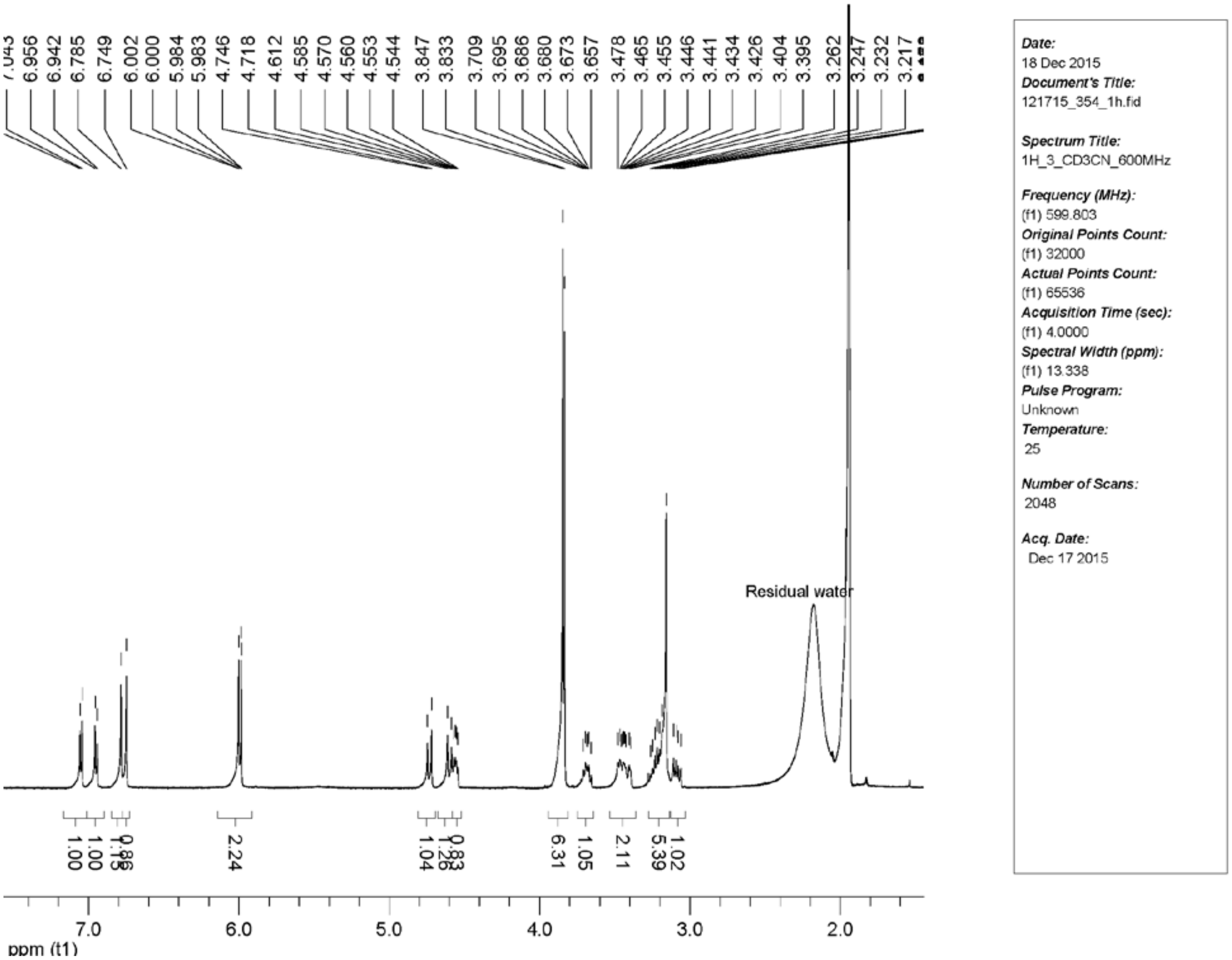
Residual water
ppm (t1)
Date:
18 Dec 2015
Document's Title:
121715_354_1h.fid
Spectrum Title:
1H_3_CD3CN_600MHz
Frequency (MHz):
(f1) 599.803
Original Points Count:
(f1) 32000
Actual Points Count:
(f1) 65536
Acquisition Time (sec):
(f1) 4.0000
Spectral Width (ppm):
(f1) 13.338
Pulse Program:
Unknown
Temperature:
25
Number of Scans:
2048
Acq. Date:
Dec 17 2015

b

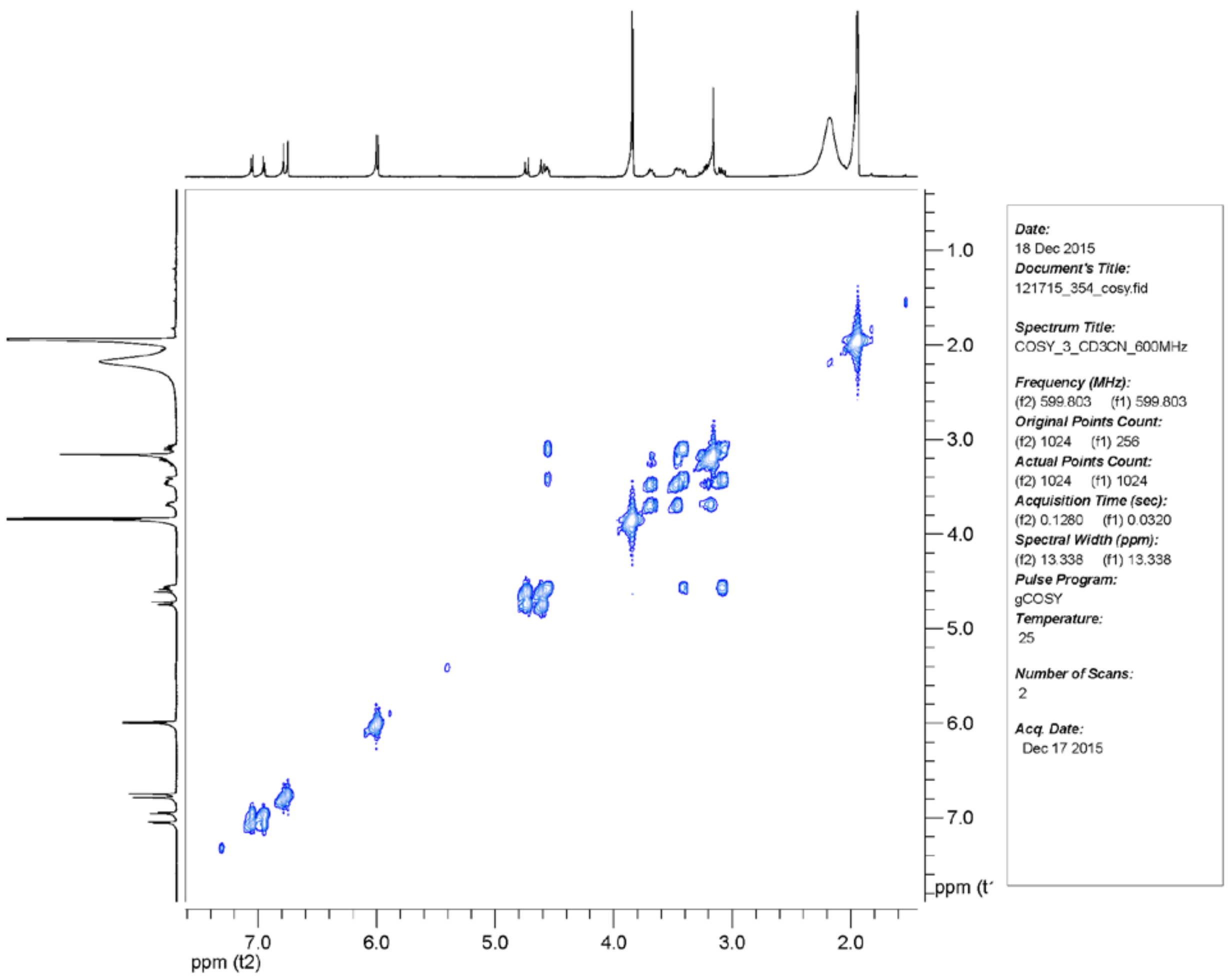

1.0
2.0
3.0
4.0
5.0
6.0
7.0
ppm (t1)
7.0
6.0
5.0
4.0
3.0
2.0
ppm (t2)
Date:
18 Dec 2015
Document's Title:
121715_354_cosy.fid
Spectrum Title:
COSY_3_CD3CN_600MHz
Frequency (MHz):
(f2) 599.803 (f1) 599.803
Original Points Count:
(f2) 1024 (f1) 256
Actual Points Count:
(f2) 1024 (f1) 1024
Acquisition Time (sec):
(f2) 0.1280 (f1) 0.0320
Spectral Width (ppm):
(f2) 13.338 (f1) 13.338
Pulse Program:
gCOSY
Temperature:
25
Number of Scans:
2
Acq. Date:
Dec 17 2015

c

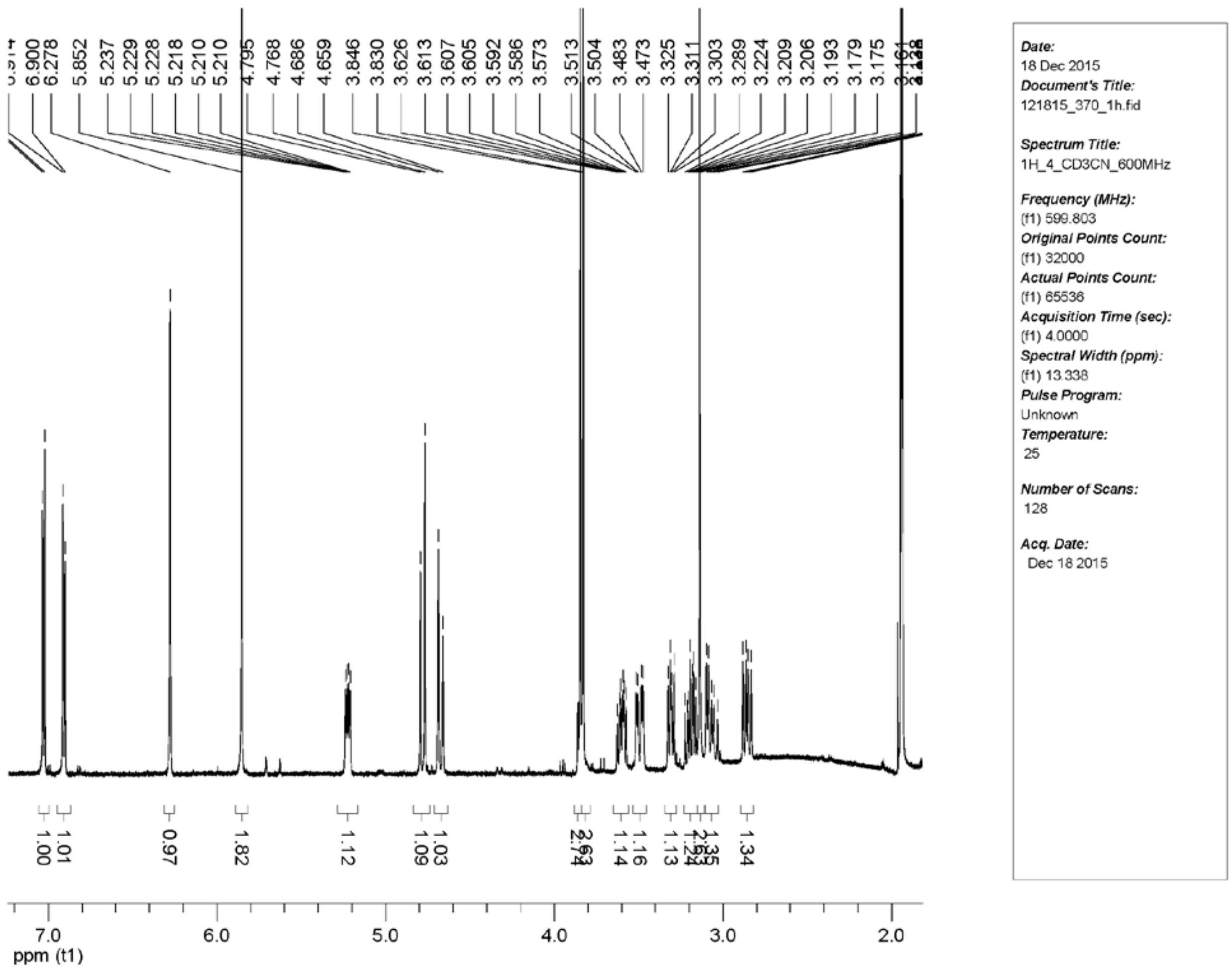
Date:
18 Dec 2015
Document's Title:
121815_370_1h.fid
Spectrum Title:
1H_4_CD3CN_600MHz
Frequency (MHz):
(f1) 599.803
Original Points Count:
(f1) 32000
Actual Points Count:
(f1) 65536
Acquisition Time (sec):
(f1) 4.0000
Spectral Width (ppm):
(f1) 13.338
Pulse Program:
Unknown
Temperature:
25
Number of Scans:
128
Acq. Date:
Dec 18 2015
ppm (t1)
7.0
6.0
5.0
4.0
3.0
2.0

d

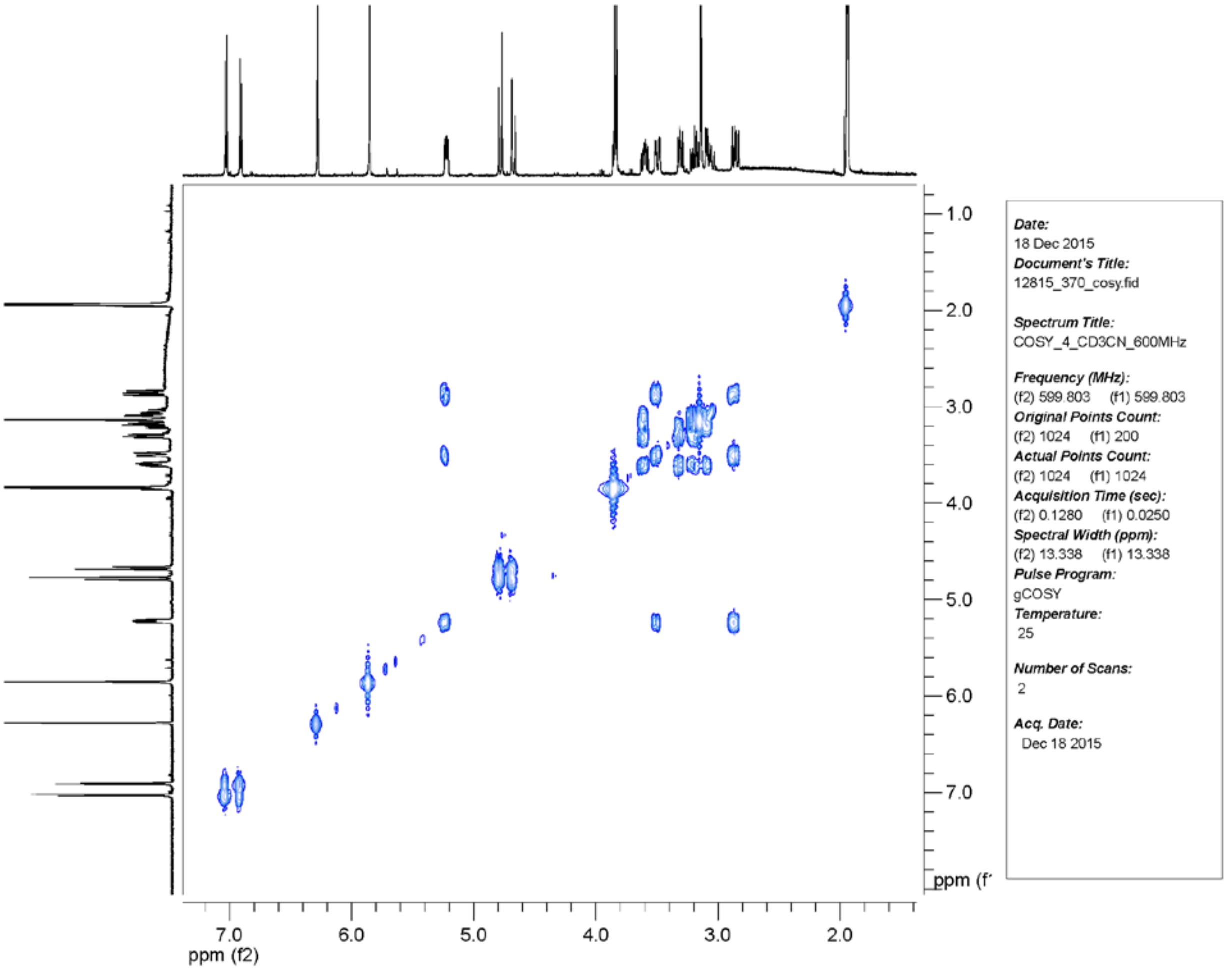

Supplementary Figure 2. NMR Spectra Data of N-methylcanadine, 3 & 1-hydroxy-N-methylcanadine, 4

(a) ^{1}H and (**b**) COSY NMR spectrum of **3**; **(c)** ^{1}H and (**d**) COSY NMR spectrum of **4**. The NMR spectra were obtained at 600 MHz and recorded in CD_3CN.

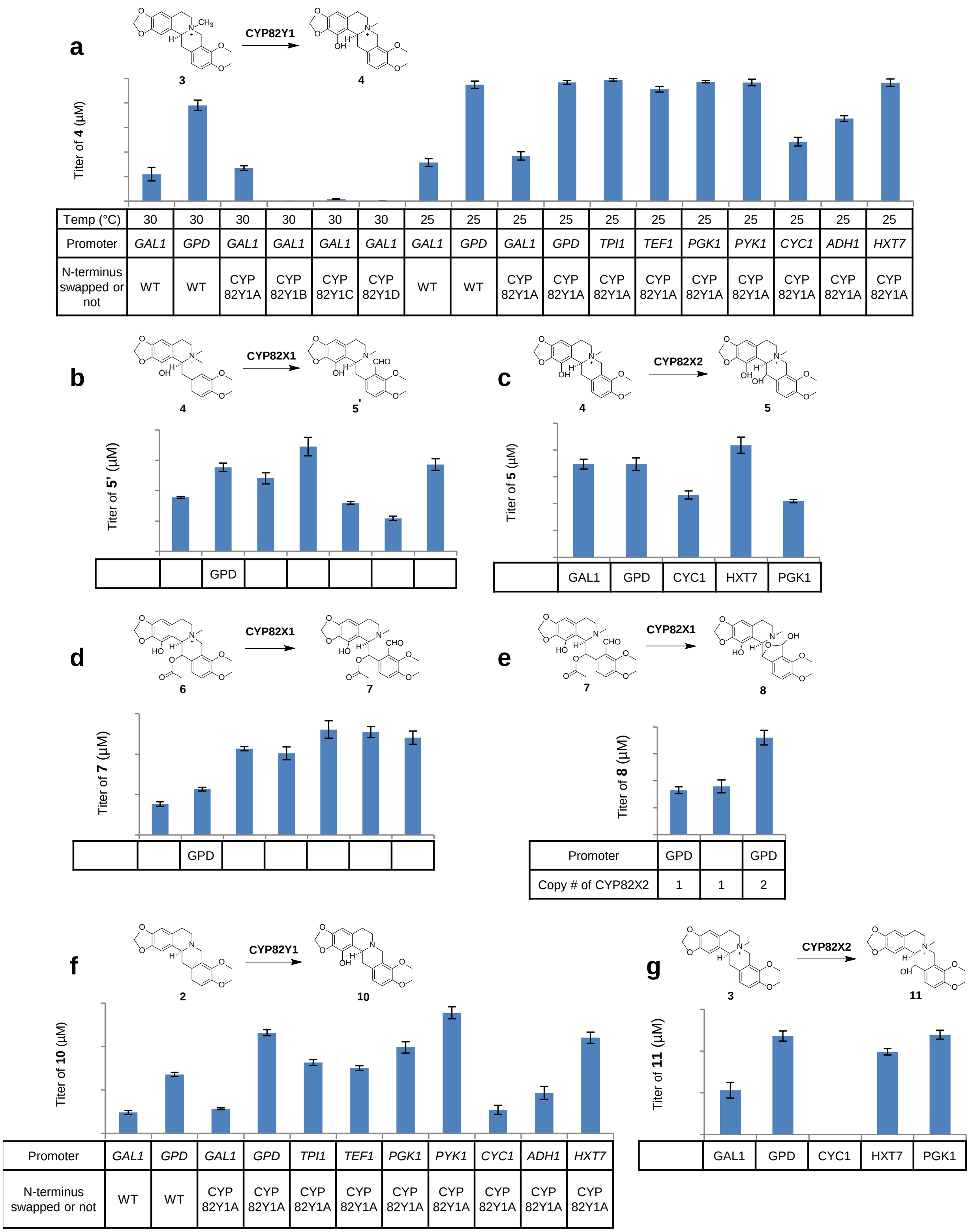

Supplementary Figure 3. Optimization of CYP82Y1, CYP82X1, and CYP82X2 activities for increased production of 4, 10, 5', 5, 7, and 11

(a) Optimization of CYP82Y1 activity for the synthesis of **4** through varying the N-terminal tag, promoter, and growth temperature. **(b)** Optimization of CYP82X1 activity for the synthesis of **5'** through varying the promoter at 25°C. **(c)** Optimization of CYP82X2 activity for the synthesis of **5** through varying the promoter at 25°C. **(d)** Optimization of CYP82X1 activity for the synthesis of **7** through varying the promoter at 25°C. **(e)** Activity of PsCXE1 for the synthesis of **8** downstream of different strong promoters (*GPD*, *PYK1*). **(f)** Optimization of CYP82Y1 activity for the synthesis of **10** through varying the N-terminal tag and promoter at 25°C. **(g)** Optimization of CYP82X2 activity for the synthesis of **11** through varying the promoter at 25°C. Data compares titers from the strain harboring the P450s expressed from the *GAL1* promoter (at 25 or 30°C) to the optimized strain harboring the best identified expression strategy for each enzyme at 25°C or 30°C. For all assays, yeast strains were fed 250 µM racemic **2** and grown in defined medium for 72 hours. Metabolites in the culture medium were analyzed and quantified by LC-MS/MS. Bars represent mean values ± 1 s.d. of three biological replicates, and the error bars represent the standard deviation of the replicates. CYP82Y1A, MSH N-terminus swapped CYP82Y1; CYP82Y1B, L14D N-terminus swapped CYP82Y1; CYP82Y1C, CmCPR N-terminus swapped CYP82Y1; CYP82Y1D, EcCFS N-terminus swapped CYP82Y1.

a

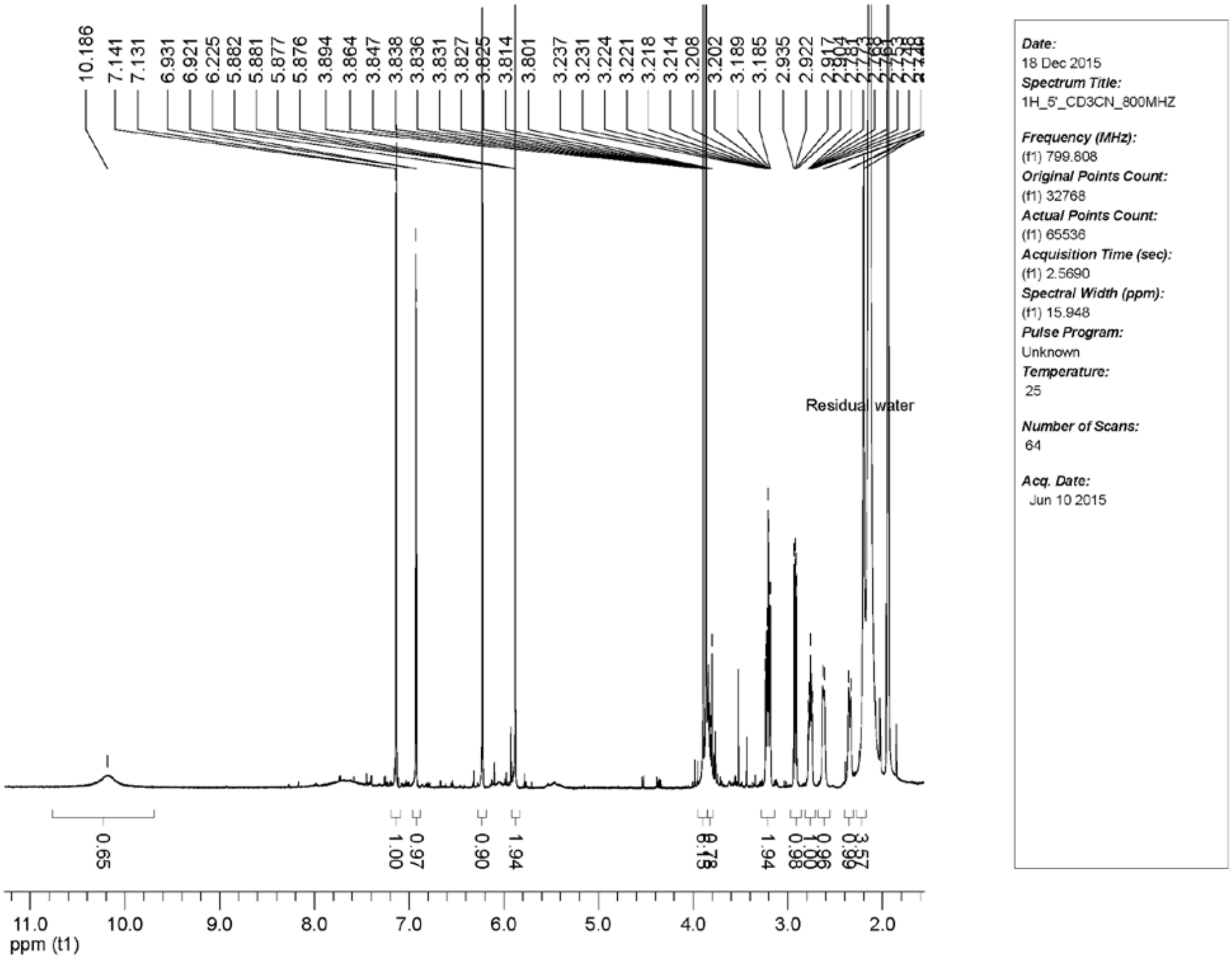
10.186
7.141
7.131
6.931
6.921
6.225
5.882
5.881
5.877
5.876
3.894
3.864
3.847
3.838
3.836
3.831
3.827
3.825
3.814
3.801
3.237
3.231
3.224
3.221
3.218
3.214
3.208
3.202
3.189
3.185
2.935
2.922
2.917
Residual water
0.65
1.00
0.97
0.90
1.94
1.94
0.98
3.57
11.0
10.0
9.0
8.0
7.0
6.0
5.0
4.0
3.0
2.0
ppm (t1)
Date:
18 Dec 2015
Spectrum Title:
1H_5'_CD3CN_800MHZ
Frequency (MHz):
(f1) 799.808
Original Points Count:
(f1) 32768
Actual Points Count:
(f1) 65536
Acquisition Time (sec):
(f1) 2.5690
Spectral Width (ppm):
(f1) 15.948
Pulse Program:
Unknown
Temperature:
25
Number of Scans:
64
Acq. Date:
Jun 10 2015

b

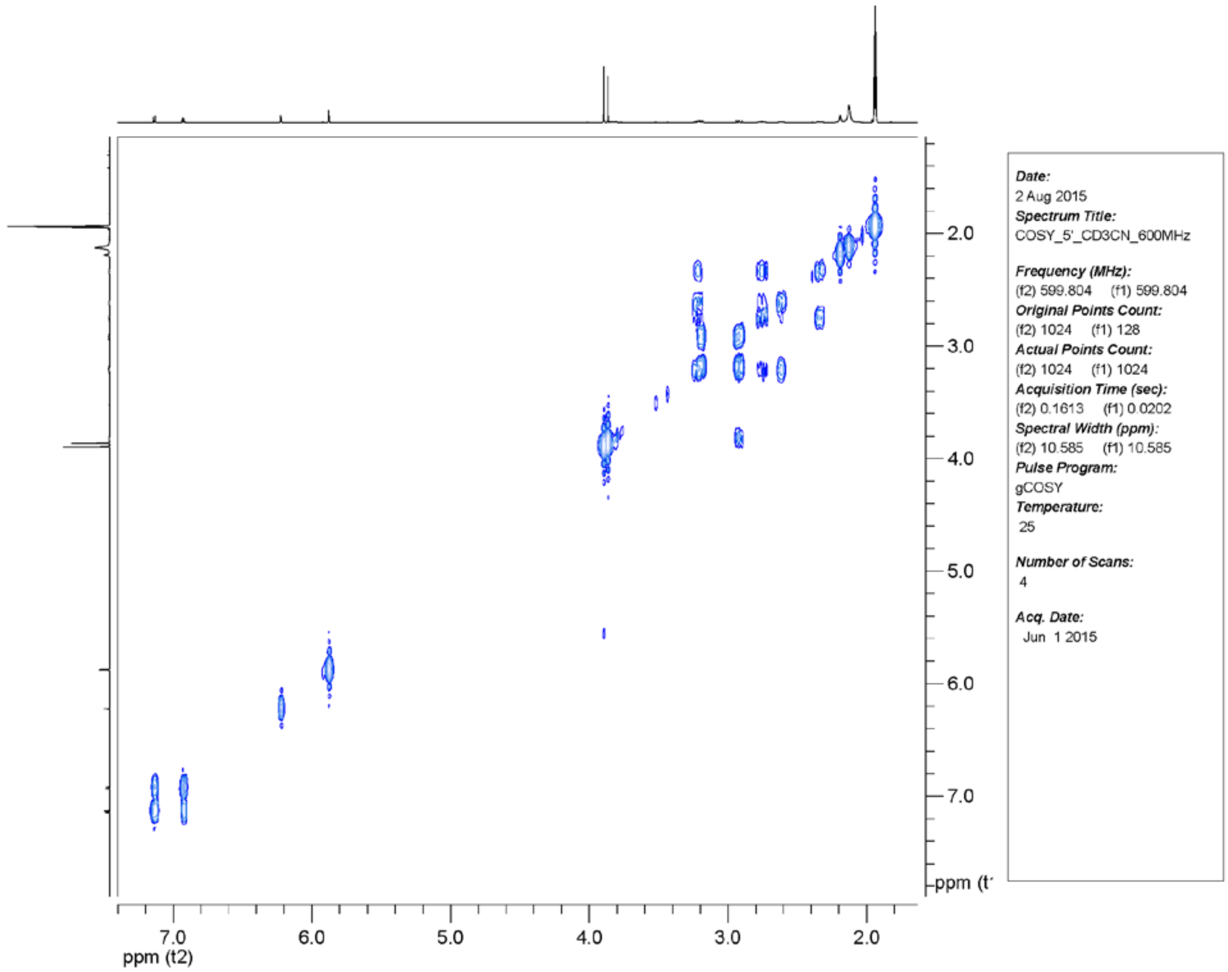

2.0
3.0
4.0
5.0
6.0
7.0
ppm (t1)
7.0
6.0
5.0
4.0
3.0
2.0
ppm (t2)
Date:
2 Aug 2015
Spectrum Title:
COSY_5'_CD3CN_600MHz
Frequency (MHz):
(f2) 599.804 (f1) 599.804
Original Points Count:
(f2) 1024 (f1) 128
Actual Points Count:
(f2) 1024 (f1) 1024
Acquisition Time (sec):
(f2) 0.1613 (f1) 0.0202
Spectral Width (ppm):
(f2) 10.585 (f1) 10.585
Pulse Program:
gCOSY
Temperature:
25
Number of Scans:
4
Acq. Date:
Jun 1 2015

c

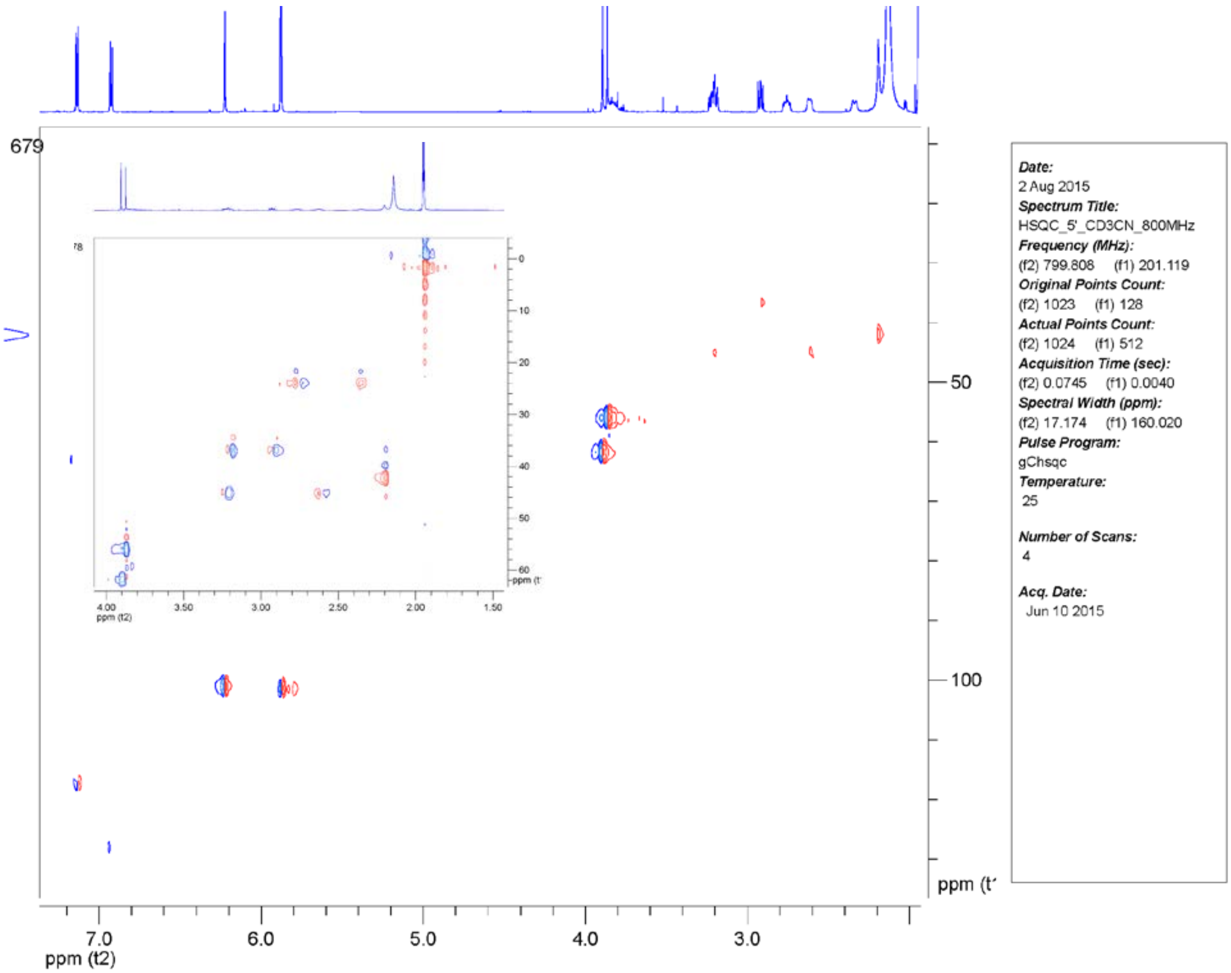

679
50
100
ppm (t1)
7.0
6.0
5.0
4.0
3.0
ppm (t2)
4.00
3.50
3.00
2.50
2.00
1.50
ppm (t2)
0
10
20
30
40
50
60
Date:
2 Aug 2015
Spectrum Title:
HSQC_5'_CD3CN_800MHz
Frequency (MHz):
(f2) 799.808 (f1) 201.119
Original Points Count:
(f2) 1023 (f1) 128
Actual Points Count:
(f2) 1024 (f1) 512
Acquisition Time (sec):
(f2) 0.0745 (f1) 0.0040
Spectral Width (ppm):
(f2) 17.174 (f1) 160.020
Pulse Program:
gChsqc
Temperature:
25
Number of Scans:
4
Acq. Date:
Jun 10 2015

d

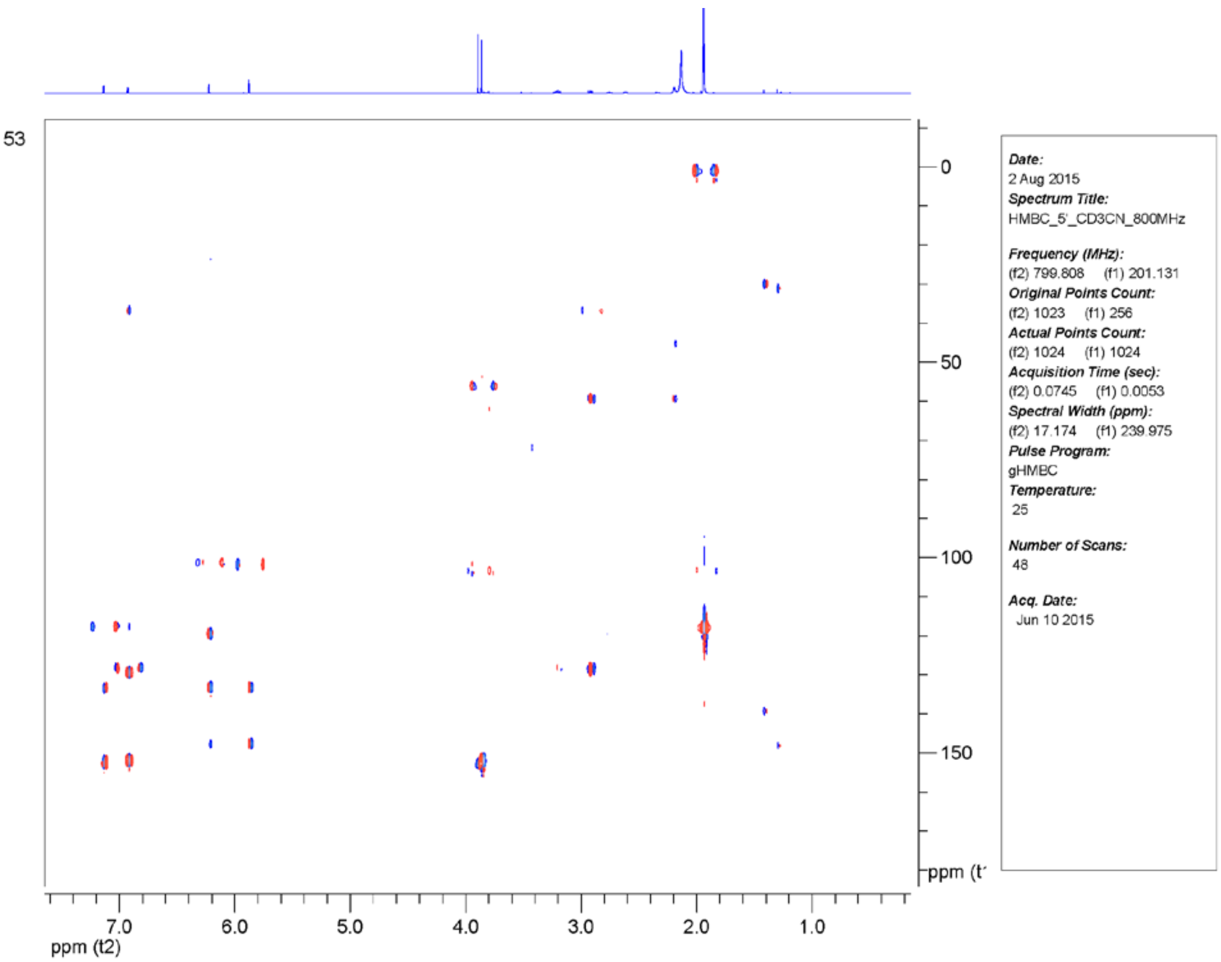

Supplementary Figure 4. NMR Spectra Data of 4'-O-Desmethylmacrantaldehyde, 5'

(a) ^{1}H NMR, (**b**) COSY, **(c)** HSQC, and (**d**) HMBC NMR spectrum of **5'**. ^{1}H, HSQC, and HMBC NMR spectra were obtained at 800 MHz, COSY spectrum was obtained at 600 MHz and recorded in CD_3CN.

a

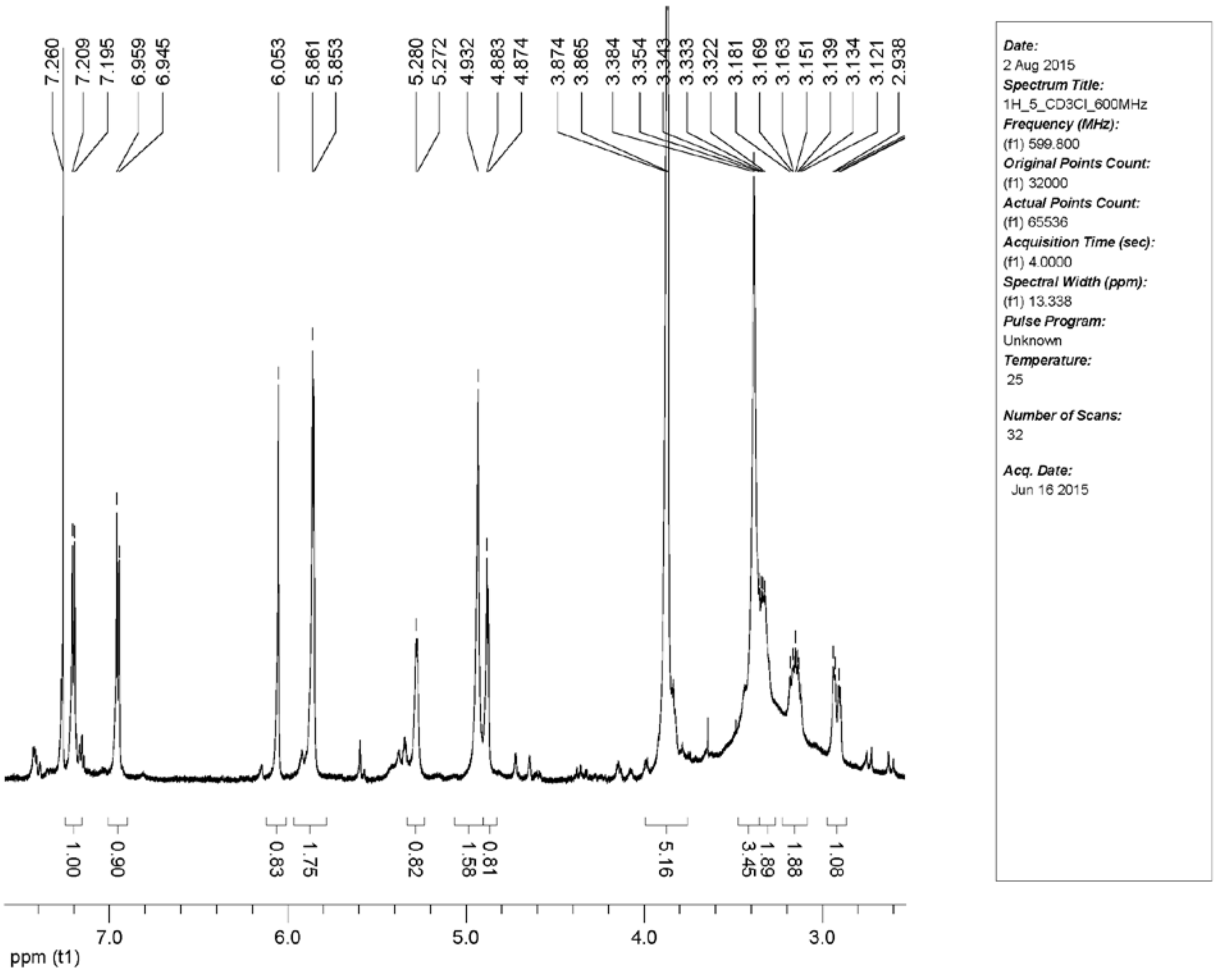
7.260
7.209
7.195
6.959
6.945
6.053
5.861
5.853
5.280
5.272
4.932
4.883
4.874
3.874
3.865
3.384
3.354
3.343
3.333
3.322
3.181
3.169
3.163
3.151
3.139
3.134
3.121
2.938
1.00
0.90
0.83
1.75
0.82
1.58
0.81
5.16
3.45
1.89
1.88
1.08
7.0
6.0
5.0
4.0
3.0
ppm (t1)
Date:
2 Aug 2015
Spectrum Title:
1H_5_CD3Cl_600MHz
Frequency (MHz):
(f1) 599.800
Original Points Count:
(f1) 32000
Actual Points Count:
(f1) 65536
Acquisition Time (sec):
(f1) 4.0000
Spectral Width (ppm):
(f1) 13.338
Pulse Program:
Unknown
Temperature:
25
Number of Scans:
32
Acq. Date:
Jun 16 2015

b

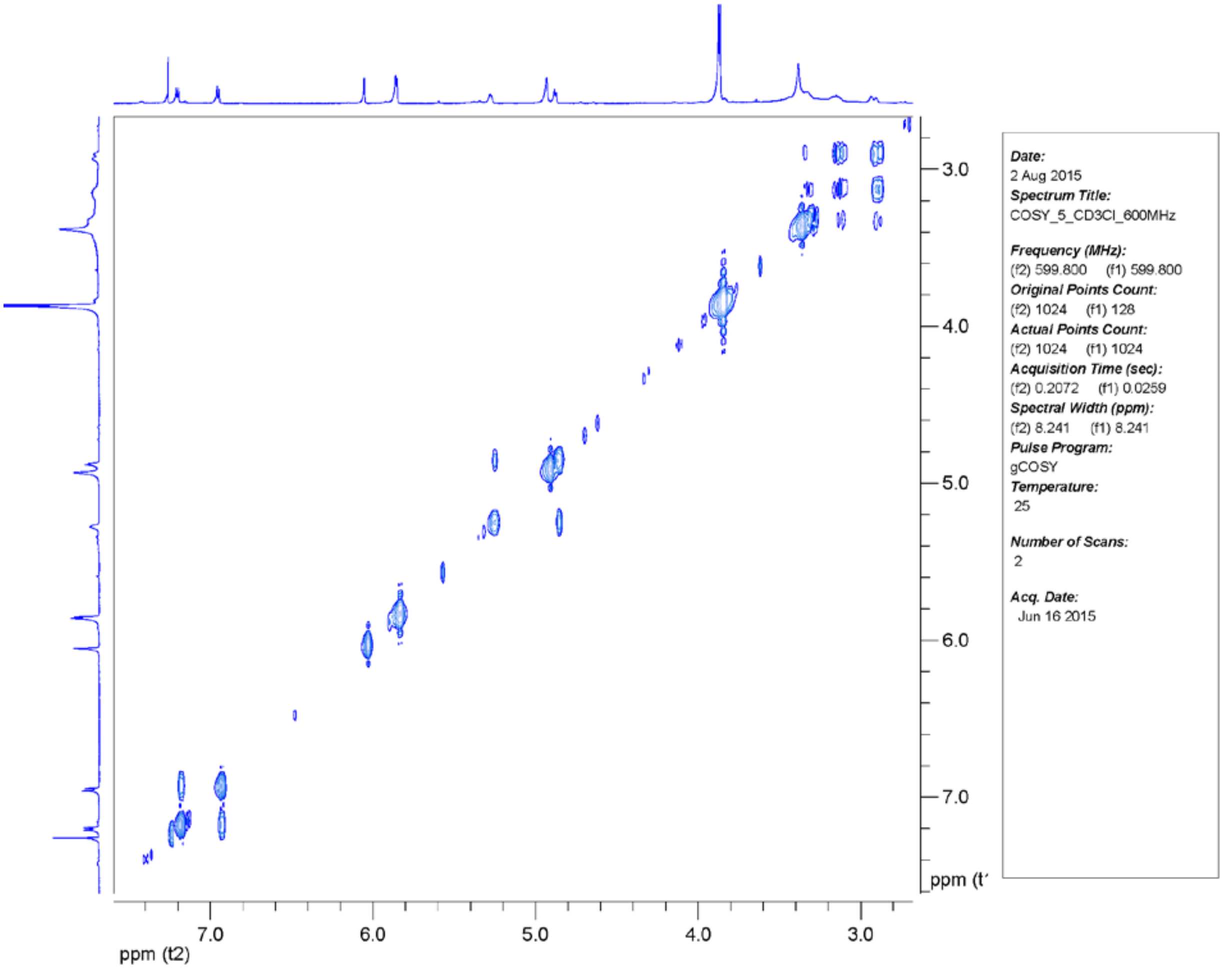

3.0
4.0
5.0
6.0
7.0
ppm (t1)
7.0
6.0
5.0
4.0
3.0
ppm (t2)
Date:
2 Aug 2015
Spectrum Title:
COSY_5_CD3Cl_600MHz
Frequency (MHz):
(f2) 599.800 (f1) 599.800
Original Points Count:
(f2) 1024 (f1) 128
Actual Points Count:
(f2) 1024 (f1) 1024
Acquisition Time (sec):
(f2) 0.2072 (f1) 0.0259
Spectral Width (ppm):
(f2) 8.241 (f1) 8.241
Pulse Program:
gCOSY
Temperature:
25
Number of Scans:
2
Acq. Date:
Jun 16 2015

c

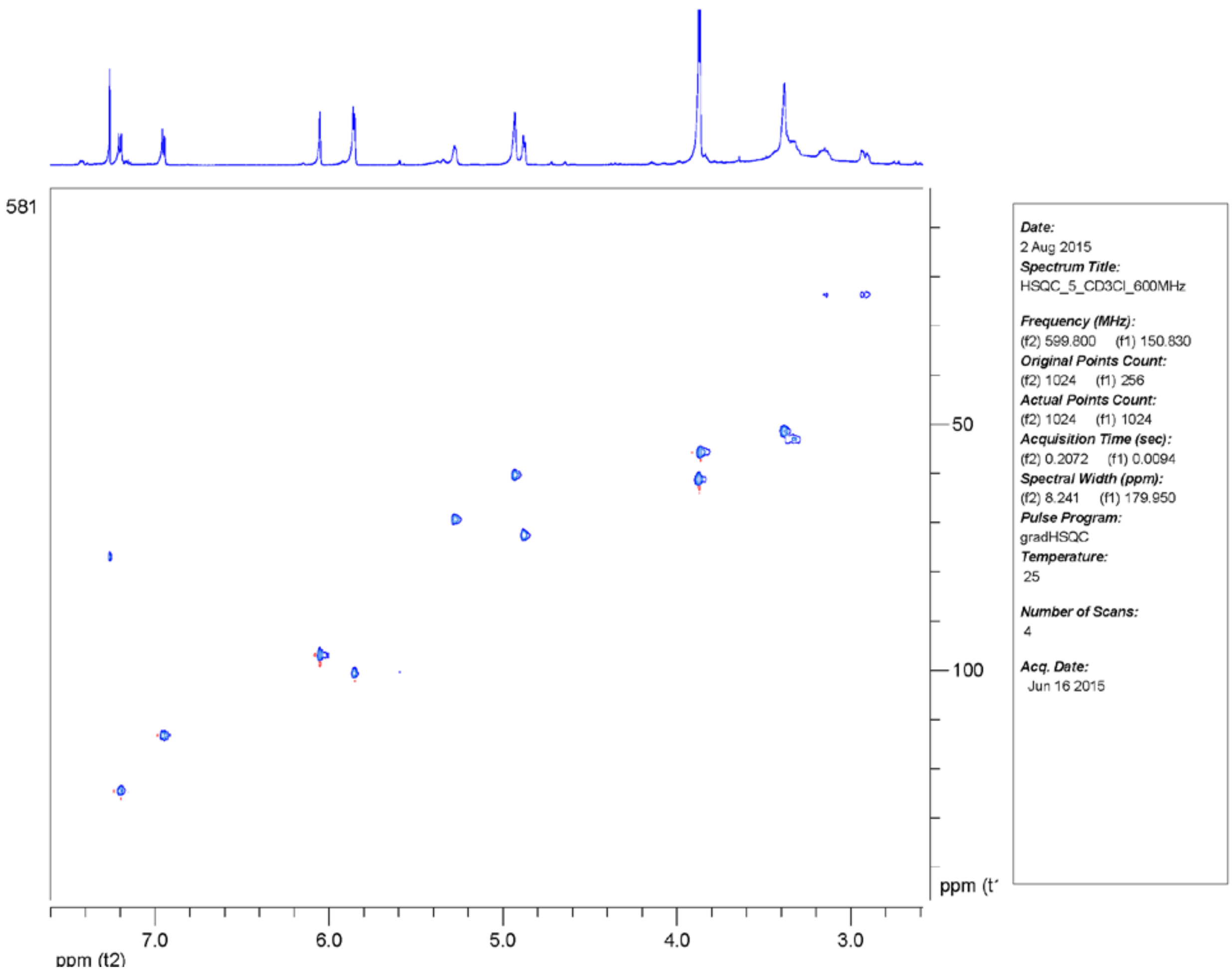
581
50
100
ppm (t
7.0
6.0
5.0
4.0
3.0
ppm (t2)
Date:
2 Aug 2015
Spectrum Title:
HSQC_5_CD3Cl_600MHz
Frequency (MHz):
(f2) 599.800 (f1) 150.830
Original Points Count:
(f2) 1024 (f1) 256
Actual Points Count:
(f2) 1024 (f1) 1024
Acquisition Time (sec):
(f2) 0.2072 (f1) 0.0094
Spectral Width (ppm):
(f2) 8.241 (f1) 179.950
Pulse Program:
gradHSQC
Temperature:
25
Number of Scans:
4
Acq. Date:
Jun 16 2015

d

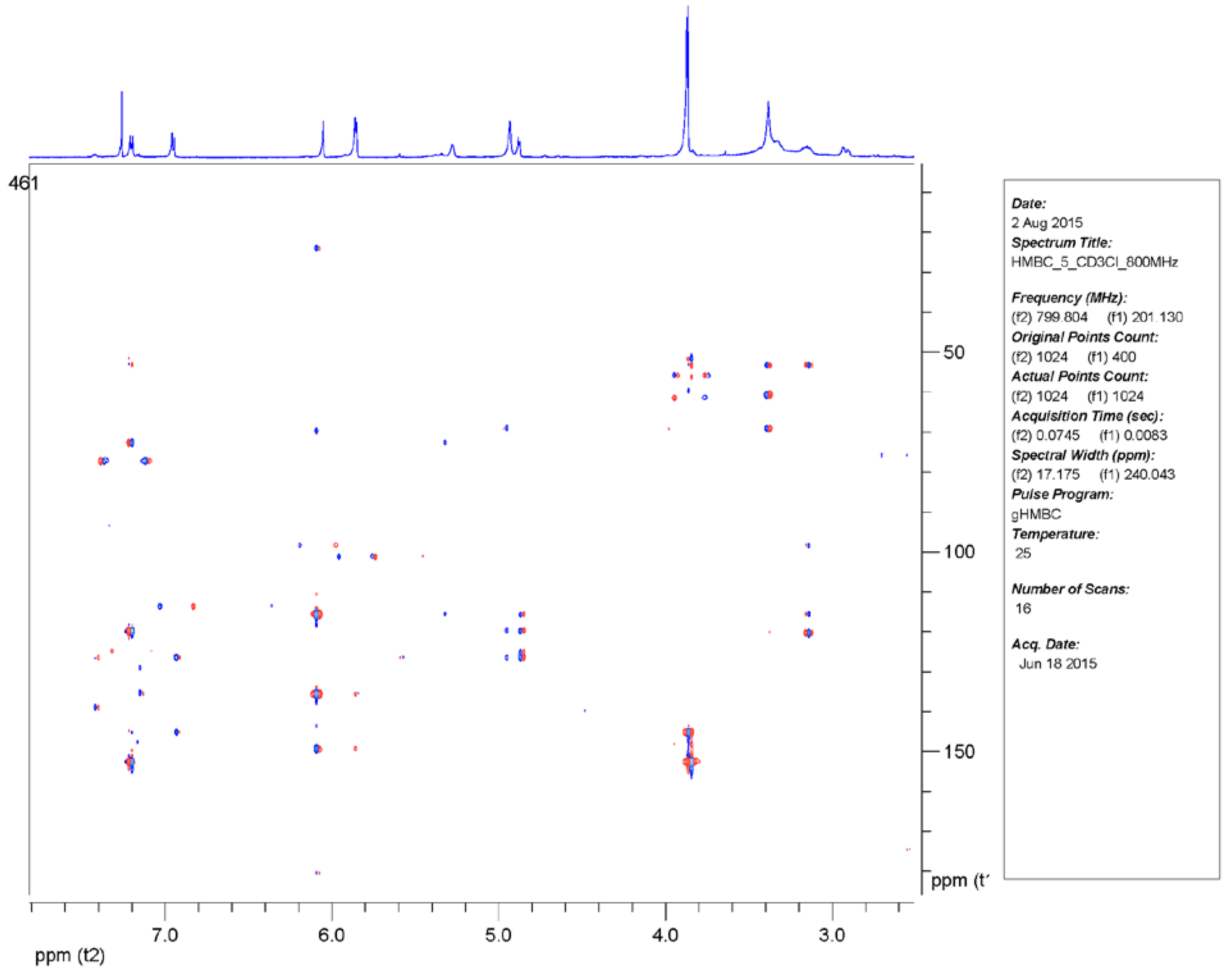

Supplementary Figure 5. NMR Spectra Data of 1,13-Dihydroxy-N-methylcanadine, 5

(a) ^{1}H NMR, (**b**) COSY, **(c)** HSQC, and (**d**) HMBC NMR spectrum of **5**. ^{1}H, COSY, and HSQC NMR spectra were obtained at 600 MHz, HMBC NMR spectrum was obtained at 800 MHz and recorded in $CDCl_3$.

a

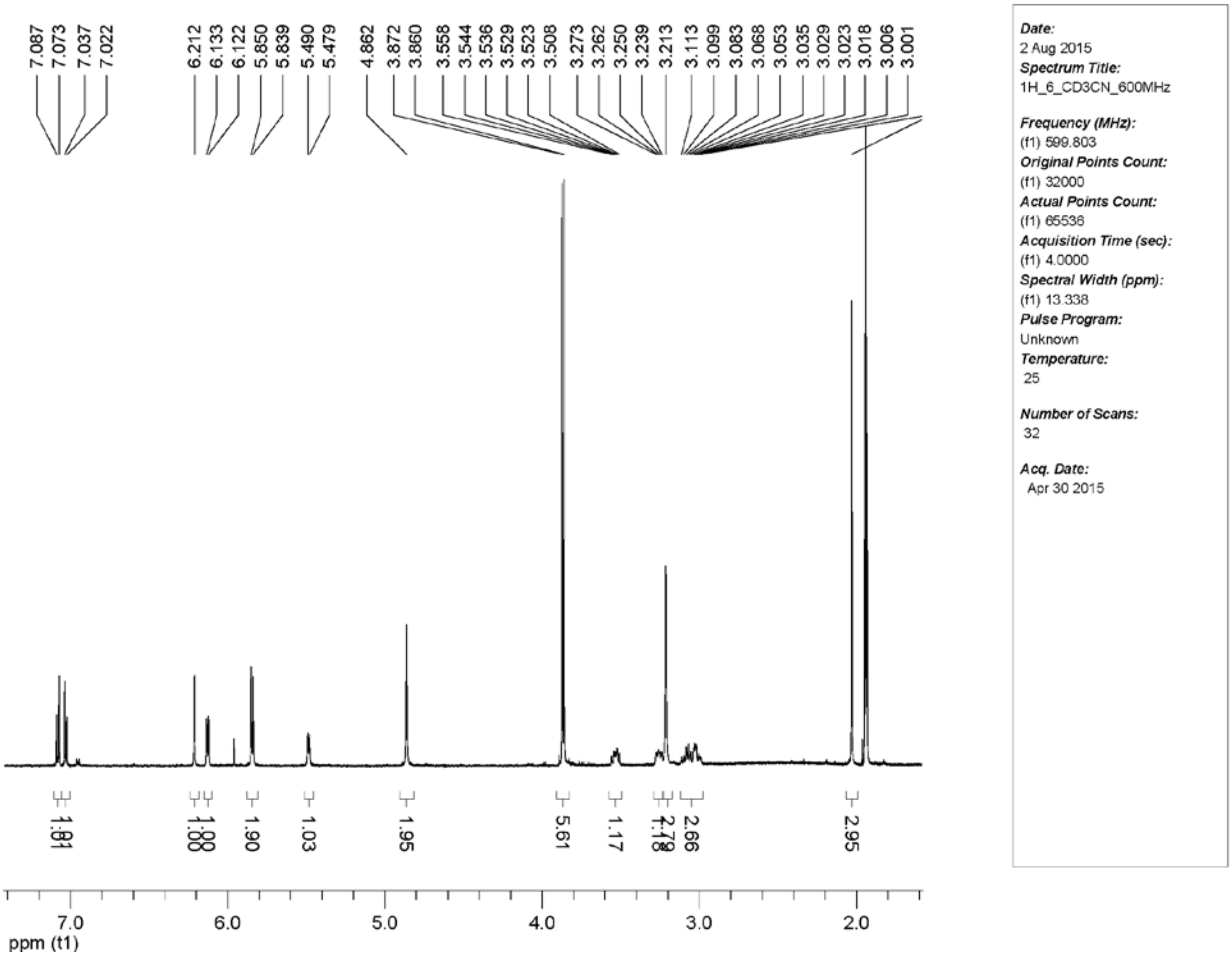

7.087
7.073
7.037
7.022
6.212
6.133
6.122
5.850
5.839
5.490
5.479
4.862
3.872
3.860
3.558
3.544
3.536
3.529
3.523
3.508
3.273
3.262
3.250
3.239
3.213
3.113
3.099
3.083
3.068
3.053
3.035
3.029
3.023
3.018
3.006
3.001
1.90
1.03
1.95
5.61
1.17
2.66
2.95
7.0
6.0
5.0
4.0
3.0
2.0
ppm (t1)
Date:
2 Aug 2015
Spectrum Title:
1H_6_CD3CN_600MHz
Frequency (MHz):
(f1) 599.803
Original Points Count:
(f1) 32000
Actual Points Count:
(f1) 65536
Acquisition Time (sec):
(f1) 4.0000
Spectral Width (ppm):
(f1) 13.338
Pulse Program:
Unknown
Temperature:
25
Number of Scans:
32
Acq. Date:
Apr 30 2015

b

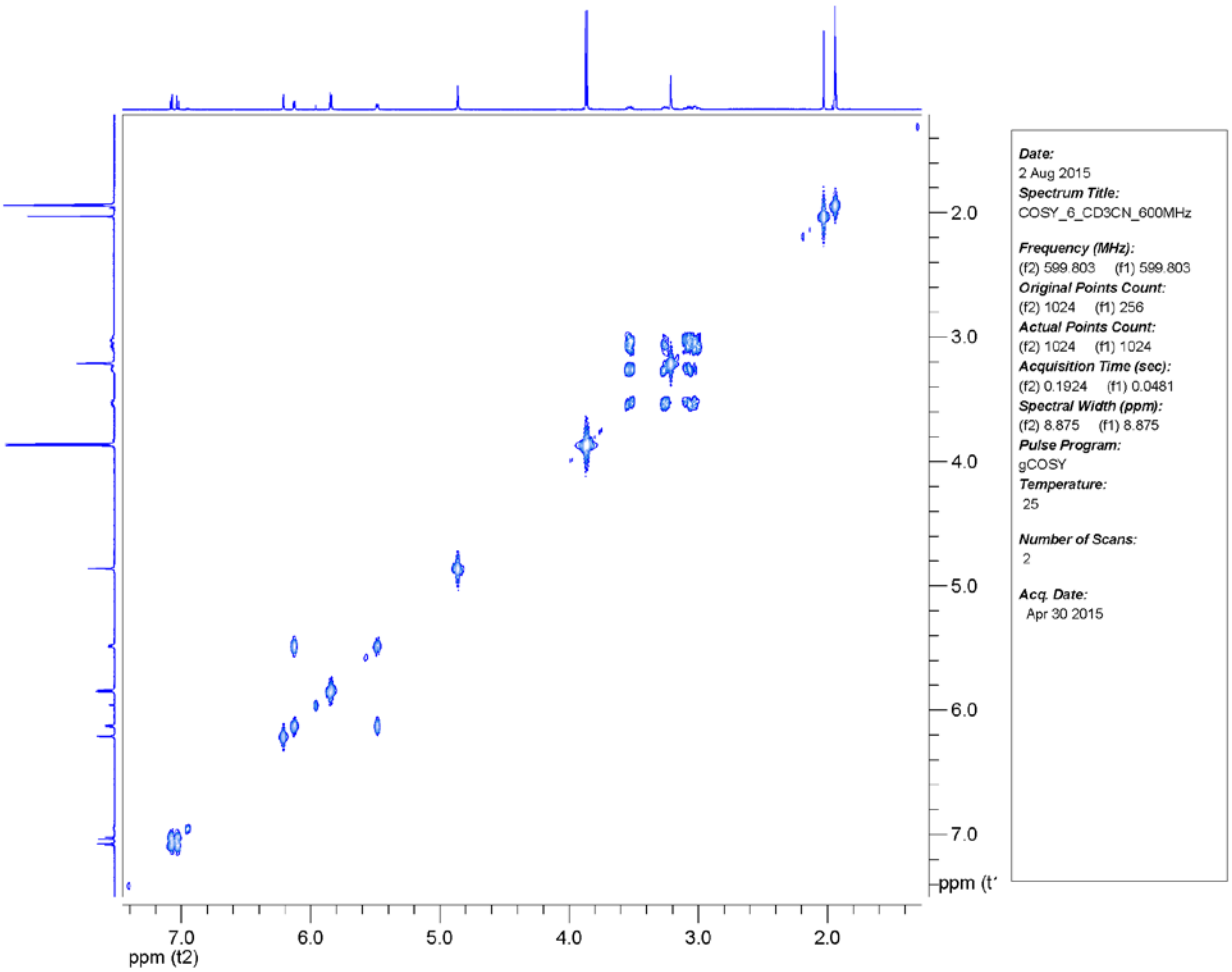
7.0
6.0
5.0
4.0
3.0
2.0
ppm (t2)
2.0
3.0
4.0
5.0
6.0
7.0
ppm (t1)
Date:
2 Aug 2015
Spectrum Title:
COSY_6_CD3CN_600MHz
Frequency (MHz):
(f2) 599.803 (f1) 599.803
Original Points Count:
(f2) 1024 (f1) 256
Actual Points Count:
(f2) 1024 (f1) 1024
Acquisition Time (sec):
(f2) 0.1924 (f1) 0.0481
Spectral Width (ppm):
(f2) 8.875 (f1) 8.875
Pulse Program:
gCOSY
Temperature:
25
Number of Scans:
2
Acq. Date:
Apr 30 2015

c

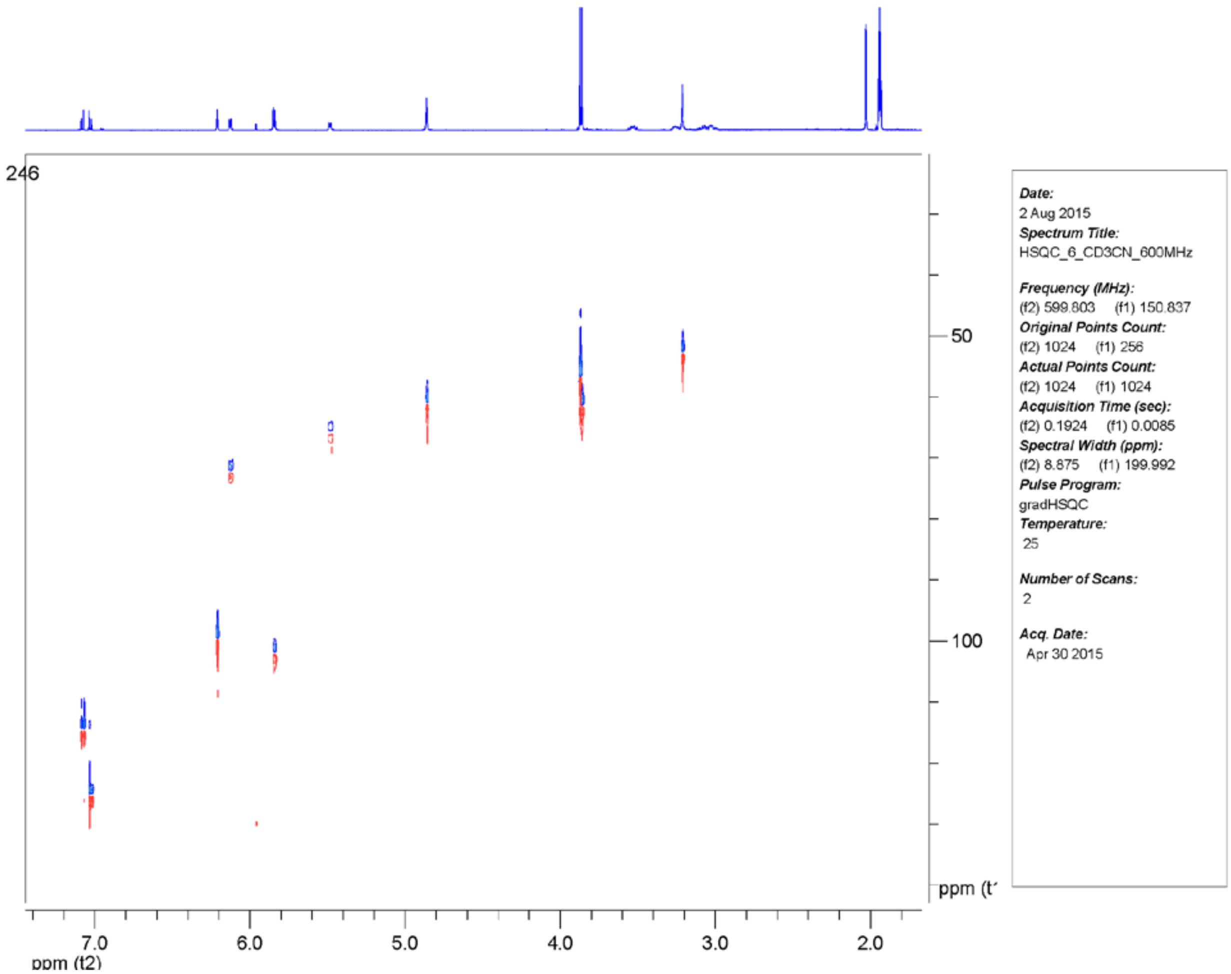
246
50
100
ppm (t1)
7.0
6.0
5.0
4.0
3.0
2.0
ppm (t2)
Date:
2 Aug 2015
Spectrum Title:
HSQC_6_CD3CN_600MHz
Frequency (MHz):
(f2) 599.803 (f1) 150.837
Original Points Count:
(f2) 1024 (f1) 256
Actual Points Count:
(f2) 1024 (f1) 1024
Acquisition Time (sec):
(f2) 0.1924 (f1) 0.0085
Spectral Width (ppm):
(f2) 8.875 (f1) 199.992
Pulse Program:
gradHSQC
Temperature:
25
Number of Scans:
2
Acq. Date:
Apr 30 2015

d

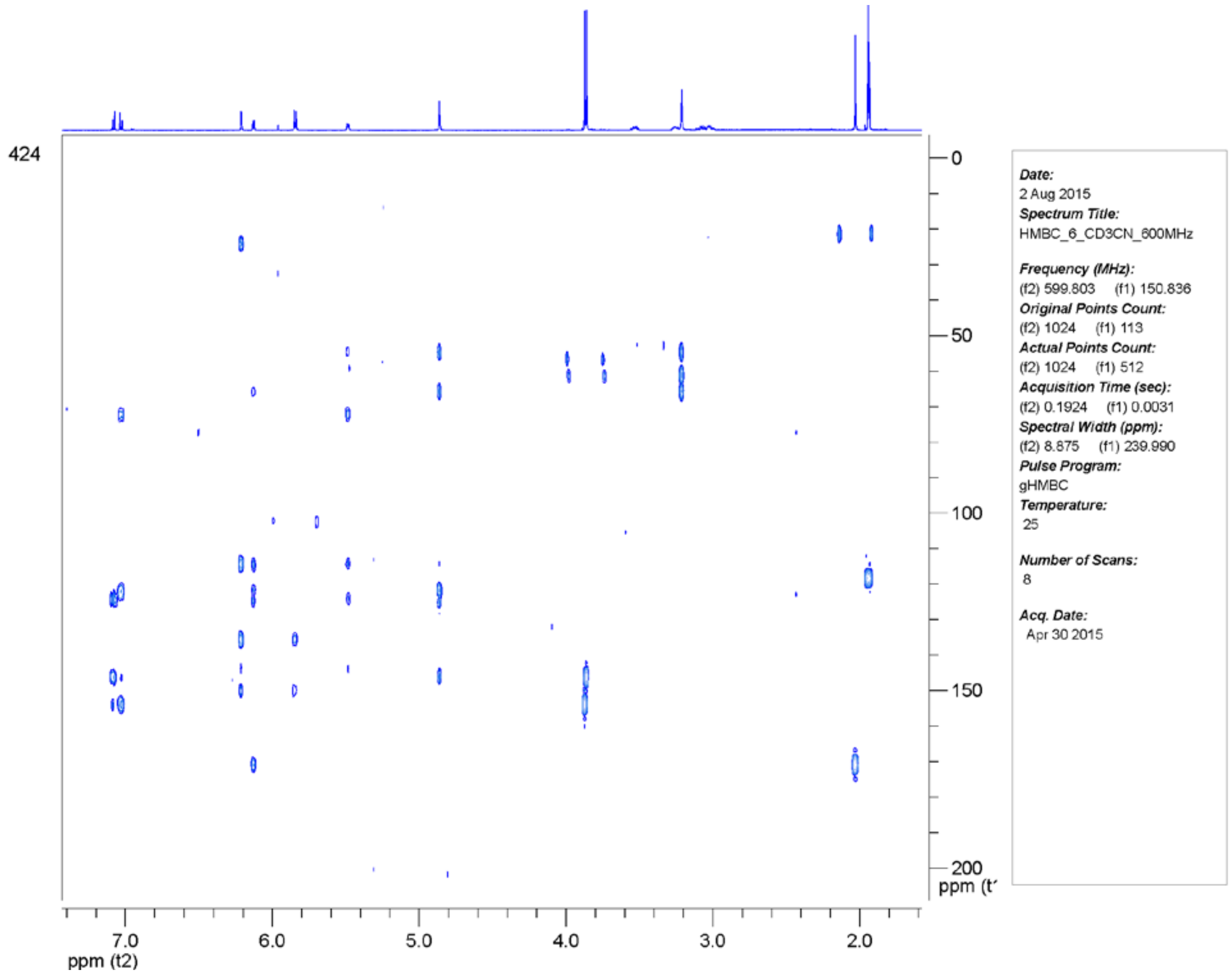

Supplementary Figure 6. NMR Spectra Data of 1-hydroxy-13-O-acetyl-N-methylcanadine, 6

(a) ^{1}H NMR, (**b**) COSY, **(c)** HSQC, and (**d**) HMBC NMR spectrum of **6** in formate form. ^{1}H, COSY, HSQC, and HMBC NMR spectra were obtained at 600 MHz and recorded in CD_3CN.

a

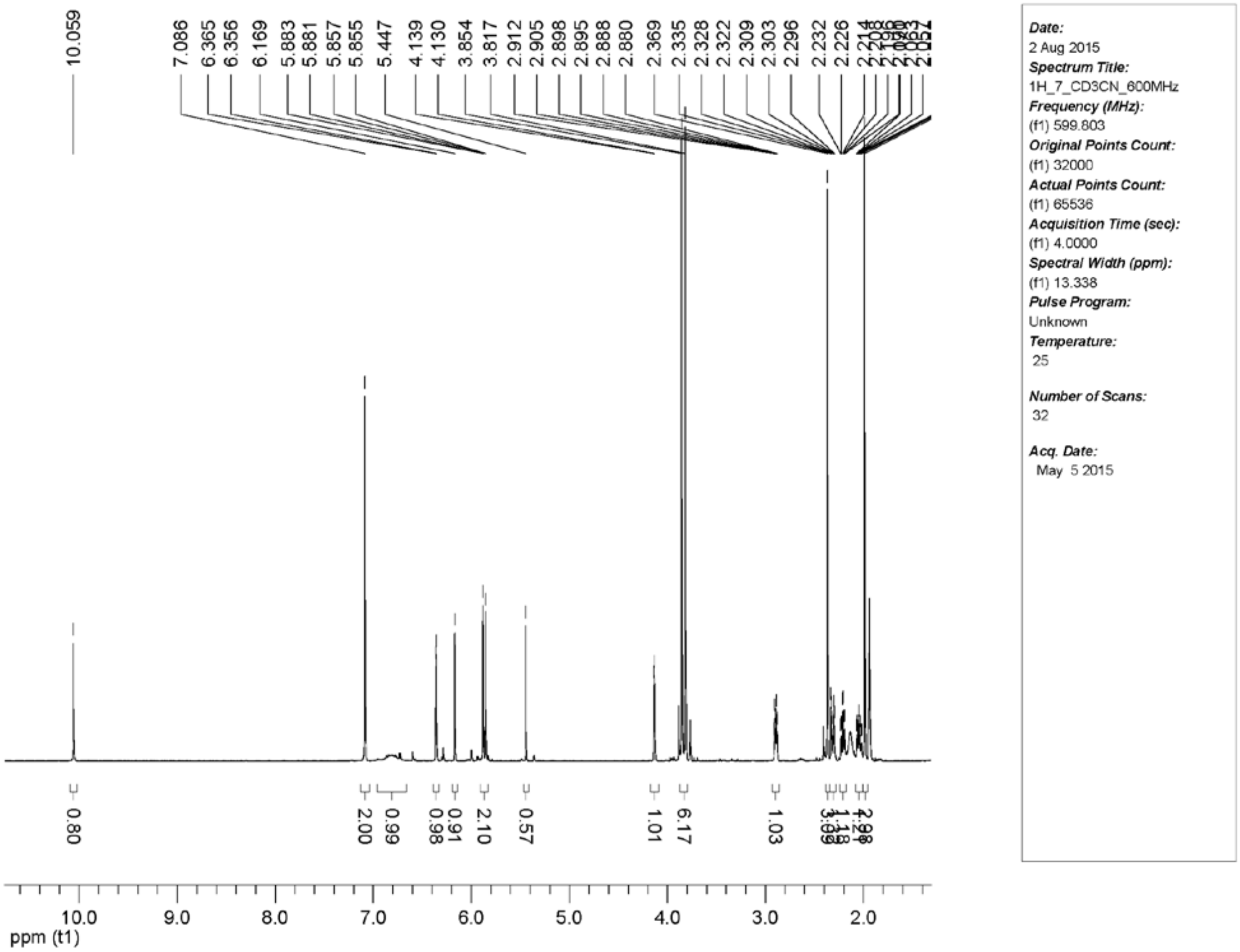

10.059
7.086
6.365
6.356
6.169
5.883
5.881
5.857
5.855
5.447
4.139
4.130
3.854
3.817
2.912
2.905
2.898
2.895
2.888
2.880
2.369
2.335
2.328
2.322
2.309
2.303
2.296
2.232
2.226
0.80
2.00
0.99
0.98
0.91
2.10
0.57
1.01
6.17
1.03
10.0
9.0
8.0
7.0
6.0
5.0
4.0
3.0
2.0
ppm (t1)
Date:
2 Aug 2015
Spectrum Title:
1H_7_CD3CN_600MHz
Frequency (MHz):
(f1) 599.803
Original Points Count:
(f1) 32000
Actual Points Count:
(f1) 65536
Acquisition Time (sec):
(f1) 4.0000
Spectral Width (ppm):
(f1) 13.338
Pulse Program:
Unknown
Temperature:
25
Number of Scans:
32
Acq. Date:
May 5 2015

b

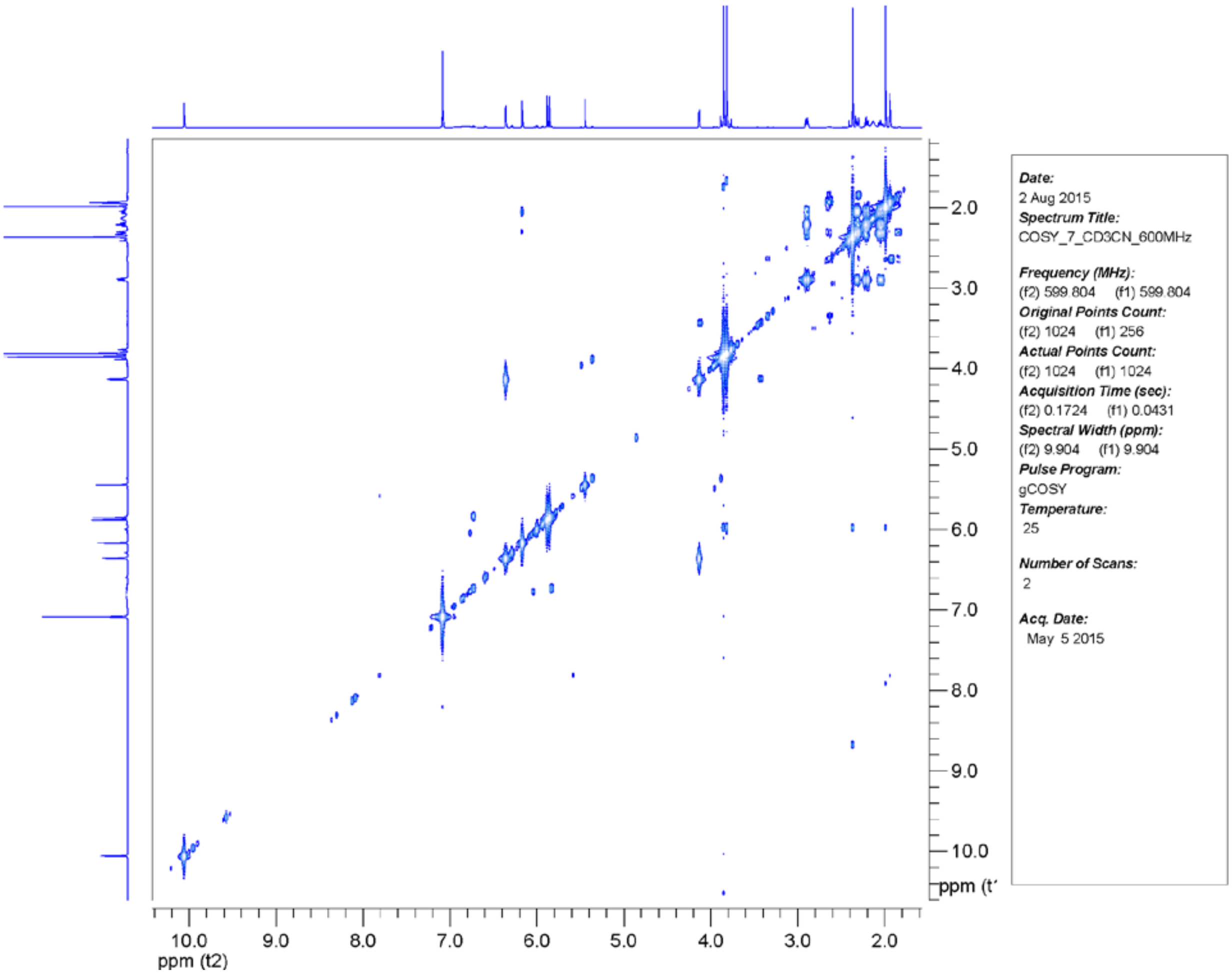

ppm (t2)
10.0
9.0
8.0
7.0
6.0
5.0
4.0
3.0
2.0
ppm (t1)
Date:
2 Aug 2015
Spectrum Title:
COSY_7_CD3CN_600MHz
Frequency (MHz):
(f2) 599.804 (f1) 599.804
Original Points Count:
(f2) 1024 (f1) 256
Actual Points Count:
(f2) 1024 (f1) 1024
Acquisition Time (sec):
(f2) 0.1724 (f1) 0.0431
Spectral Width (ppm):
(f2) 9.904 (f1) 9.904
Pulse Program:
gCOSY
Temperature:
25
Number of Scans:
2
Acq. Date:
May 5 2015

c

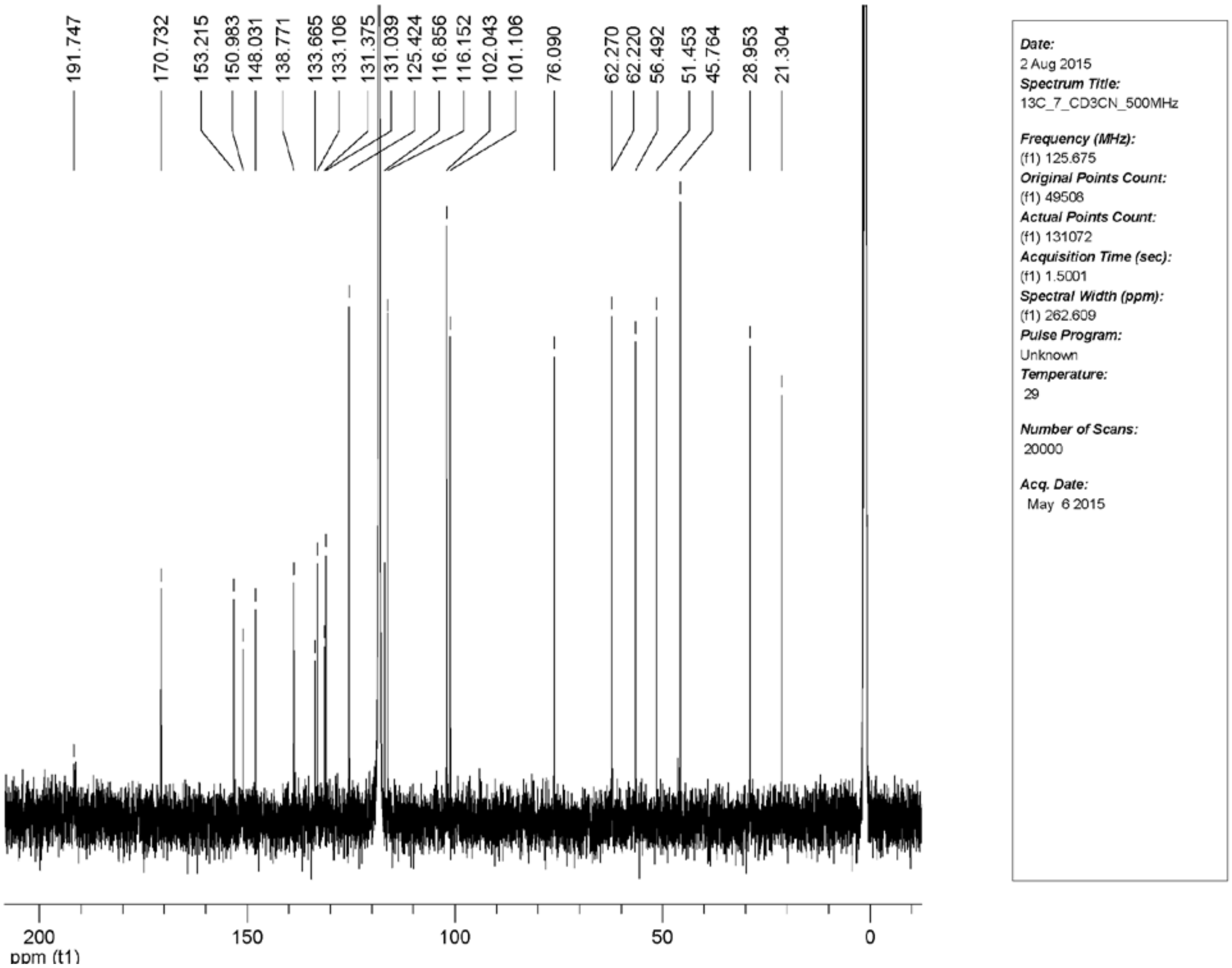
191.747
170.732
153.215
150.983
148.031
138.771
133.665
133.106
131.375
131.039
125.424
116.856
116.152
102.043
101.106
76.090
62.270
62.220
56.492
51.453
45.764
28.953
21.304
200
150
100
50
0
ppm (t1)
Date:
2 Aug 2015
Spectrum Title:
13C_7_CD3CN_500MHz
Frequency (MHz):
(f1) 125.675
Original Points Count:
(f1) 49508
Actual Points Count:
(f1) 131072
Acquisition Time (sec):
(f1) 1.5001
Spectral Width (ppm):
(f1) 262.609
Pulse Program:
Unknown
Temperature:
29
Number of Scans:
20000
Acq. Date:
May 6 2015

d

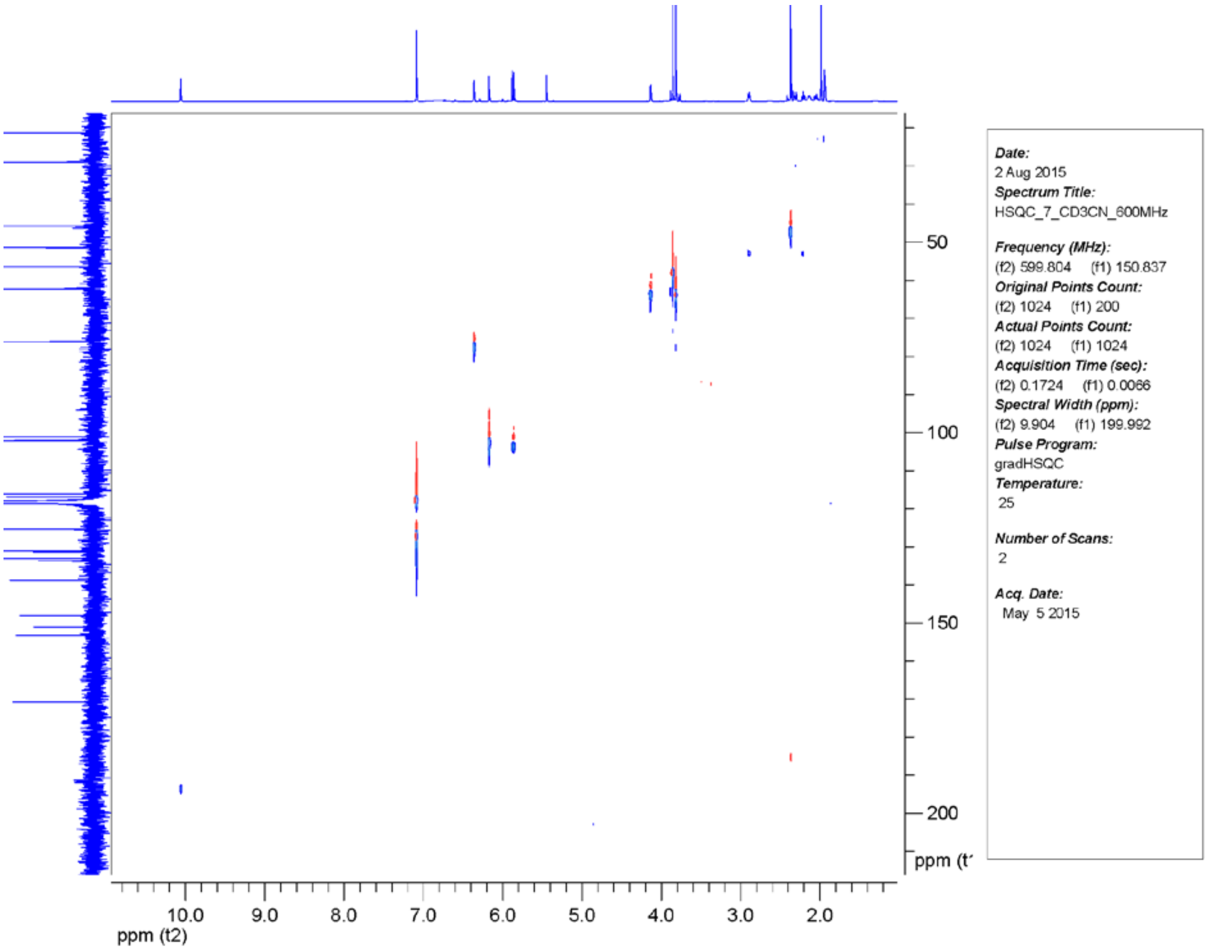

50
100
150
200
ppm (t1)
10.0
9.0
8.0
7.0
6.0
5.0
4.0
3.0
2.0
ppm (t2)
Date:
2 Aug 2015
Spectrum Title:
HSQC_7_CD3CN_600MHz
Frequency (MHz):
(f2) 599.804 (f1) 150.837
Original Points Count:
(f2) 1024 (f1) 200
Actual Points Count:
(f2) 1024 (f1) 1024
Acquisition Time (sec):
(f2) 0.1724 (f1) 0.0066
Spectral Width (ppm):
(f2) 9.904 (f1) 199.992
Pulse Program:
gradHSQC
Temperature:
25
Number of Scans:
2
Acq. Date:
May 5 2015

e

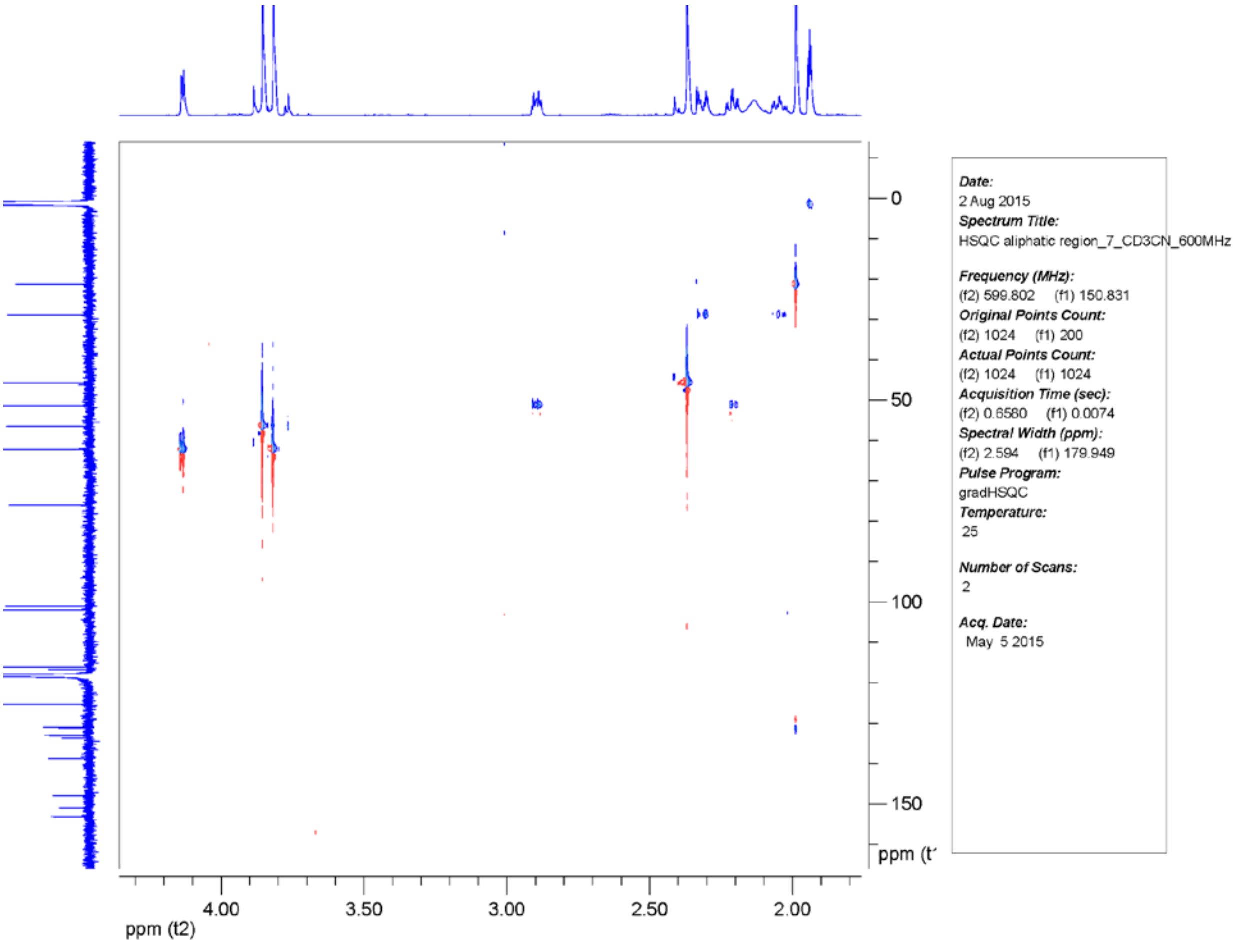

Date:
2 Aug 2015
Spectrum Title:
HSQC aliphatic region_7_CD3CN_600MHz
Frequency (MHz):
(f2) 599.802 (f1) 150.831
Original Points Count:
(f2) 1024 (f1) 200
Actual Points Count:
(f2) 1024 (f1) 1024
Acquisition Time (sec):
(f2) 0.6560 (f1) 0.0074
Spectral Width (ppm):
(f2) 2.594 (f1) 179.949
Pulse Program:
gradHSQC
Temperature:
25
Number of Scans:
2
Acq. Date:
May 5 2015
ppm (t2)
4.00
3.50
3.00
2.50
2.00
0
50
100
150

f

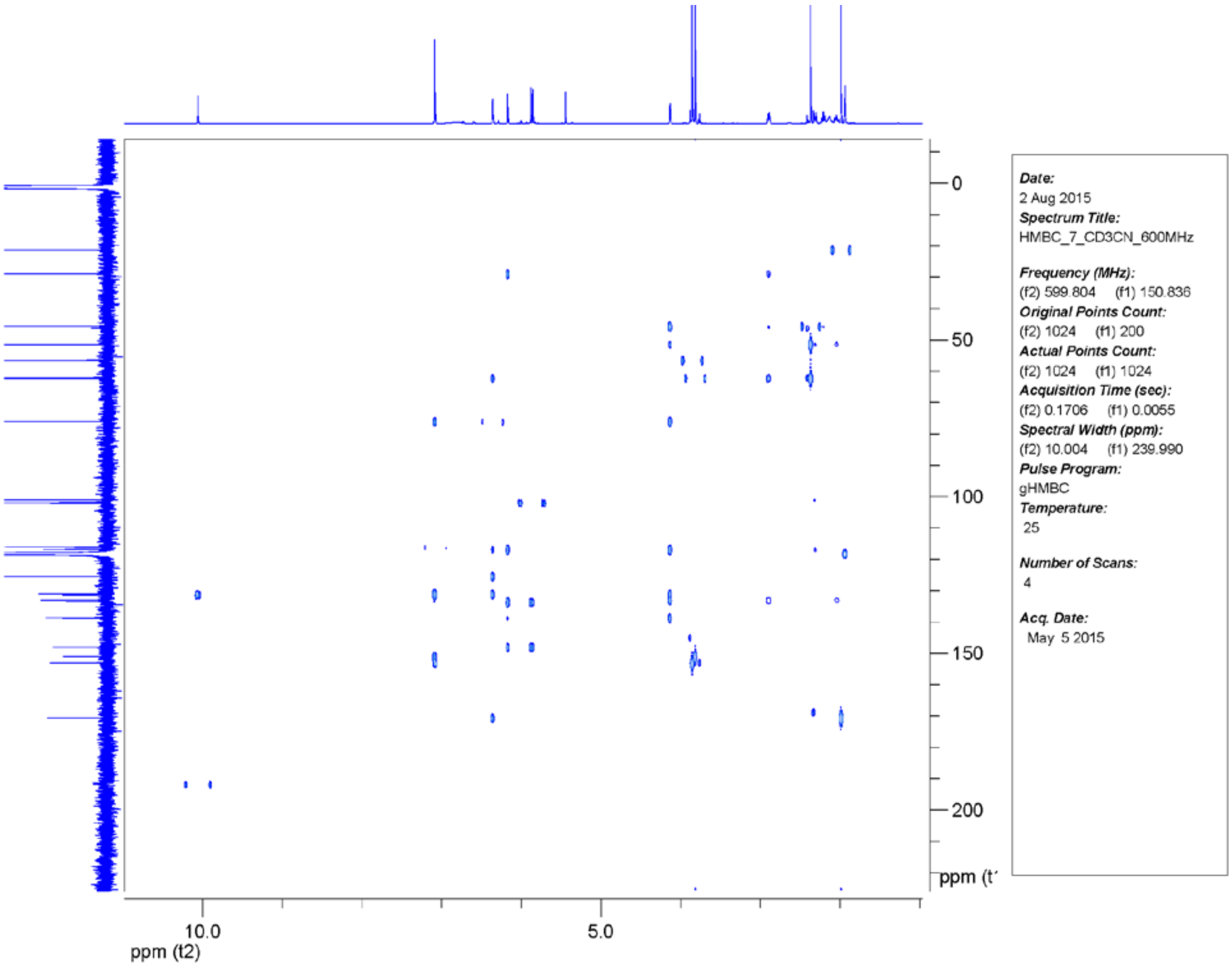

Supplementary Figure 7. NMR Spectra Data of 4'-O-Desmethyl-3-O-Acetylpapaveroxine, 7

(a) ^{1}H NMR, (**b**) COSY, **(c)** ^{13}C NMR, (**d**) HSQC spectrum, (**e**) HSQC and (**f**) HMBC spectrum in aliphatic region of **7**. ^{1}H and 2D NMR spectra were obtained at 600 MHz, ^{13}C NMR spectrum was obtained at 500 MHz and recorded in CD_3CN.

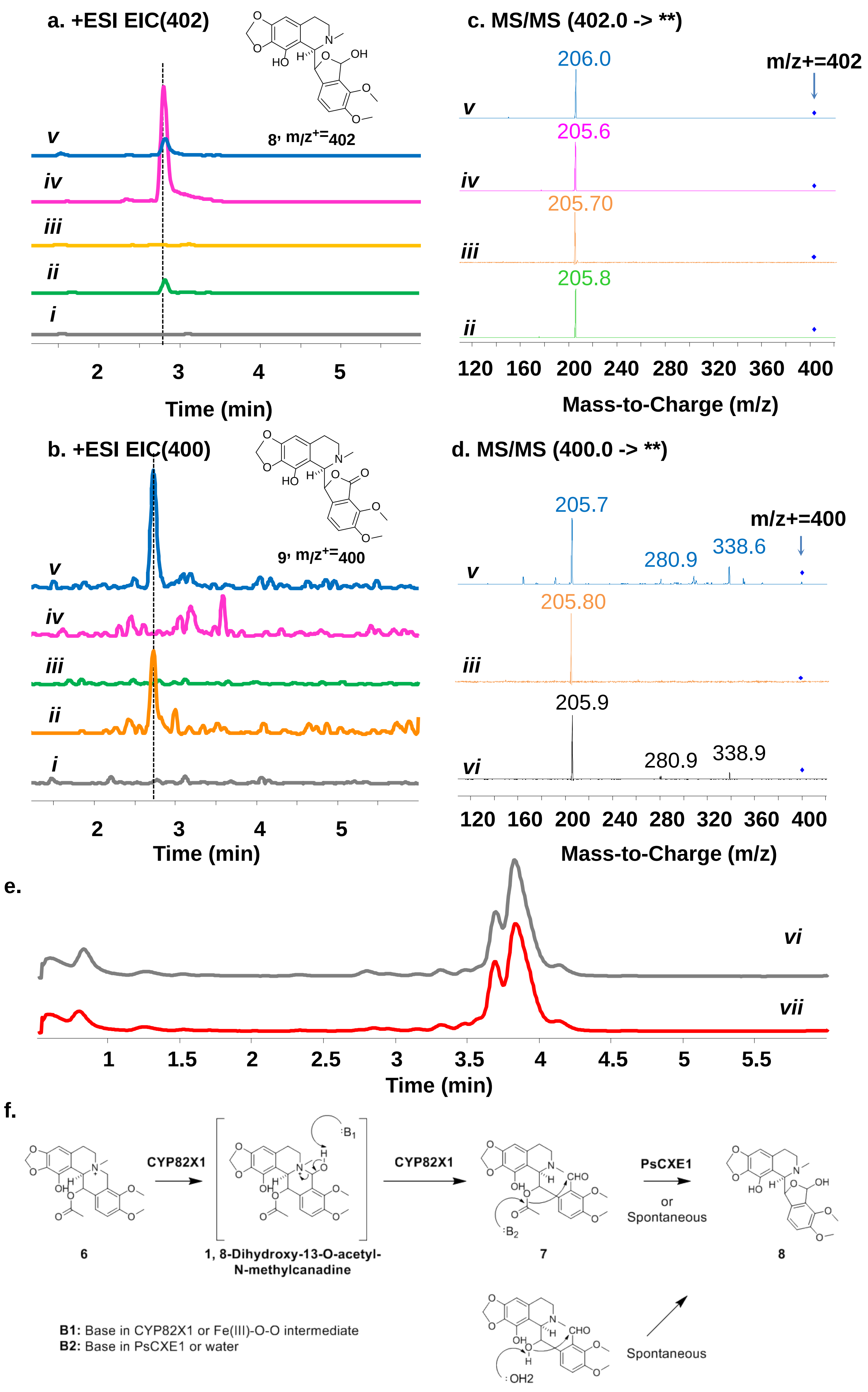

a. +ESI EIC(402)
8, m/z+=402
v
iv
iii
ii
i
2
3
4
5
Time (min)
c. MS/MS (402.0 -> **)
206.0
m/z+=402
205.6
205.70
205.8
120 160 200 240 280 320 360 400
Mass-to-Charge (m/z)
b. +ESI EIC(400)
9, m/z+=400
d. MS/MS (400.0 -> **)
205.7
m/z+=400
280.9
338.6
205.80
205.9
338.9
vi
e.
vii
1 1.5 2 2.5 3 3.5 4 4.5 5 5.5
f.
CYP82X1
:B1
PsCXE1
or
Spontaneous
6
1, 8-Dihydroxy-13-O-acetyl-N-methylcanadine
7
8
:B2
:OH2
B1: Base in CYP82X1 or Fe(III)-O-O intermediate
B2: Base in PsCXE1 or water

Supplementary Figure 8. LC-MS analysis of yeast strains engineered for the synthesis of 8 and 9, and proposed mechanism for the synthesis of 7 and 8

(a) EIC of m/z$^+$= 402 of (*i*) **5'**-producing strain, (*ii*) **5'**-producing strain expressing CYP82X2, (*iii*) **5'**-producing strain expressing CYP82X2 and PsSDR1, (*iv*) **7**-producing strain, and (*v*) **7**-producing strain expressing PsSDR1. **(b)** EIC of m/z$^+$=400 of (*i*) **5'**-producing strain, (*ii*) **5'**-producing strain expressing CYP82X2, (*iii*) **5'**-producing strain expressing CYP82X2 and PsSDR1, (*iv*) **7**-producing strain, and (*v*) **7**-producing strain expressing PsSDR1. **(c)** MS/MS spectrum of m/z$^+$ of interest at 402 at retention time = 2.8 min of (*ii*) **5'**-producing strain expressing CYP82X2, (*iii*) **5'**-producing strain expressing CYP82X2 and PsSDR1, (*iv*) **7**-producing strain, and (*v*) **7**-producing strain expressing PsSDR1. **(d)** MS/MS spectrum of m/z$^+$ of interest at 400 at retention time = 2.7 min of (*iii*) **5'**-producing strain expressing CYP82X2 and PsSDR1, (*v*) **7**-producing strain expressing PsSDR1, and (*vi*) **9**-producing strain (expressing PsTNMT, PsAT1, AtATR1, CYP82Y1, CYP82X1, CYP82X2, PsCXE1, and PsSDR1). For all assays, yeast strains were fed 250 µM racemic **2** and grown in defined medium for 72 hours. Metabolites in the culture medium were analyzed by LC-MS/MS. The identities of metabolites were confirmed by comparison to the MS2 spectra of reported standards. All traces are representative of at least 3 biological replicates for each engineered yeast strain. **(e)** Total ion chromatograph (TIC) of (*vi*) **9**-producing strain and (*vii*) **8**-producing strain. **(f)** Proposed mechanism for the synthesis of **7** and **8**. Only **7** (i.e., no 1, 8-dihydroxy-13-O-acetyl-N-methylcanadine) was detected in the yeast strain expressing PsTNMT, AtATR1, CYP82Y1, CYP82X2, PsAT1, and CYP82X1. Thus, it is likely that in addition to catalyzing C8-hydroxylation, CYP82X1 also provides the nucleophile (either residues in CYP82X1 or the Fe(III)-O-O intermediate as the attacking base) to perform the C8-hydroxy proton attraction, which promotes the N7-C8 bond cleavage to afford the formation of 4'-O-desmethyl-3-O-acetylpapaveroxine. In the absence of PsCXE1, the **7**-producing strain synthesizes more **8** than the strain producing 4'-O-desmethylpapaveroxine, **13**. The addition of PsCXE1 to the **7**-producing strain substantially increases the titer of **8** in the medium. For the synthesis of **8**, the acetyl group in **7** enables catalysis via a tetrahedral acyl intermediate rather than simple proton abstraction in **13**, resulting in more efficient ring formation subsequent to the synthesis of the unstable aldehyde.

a

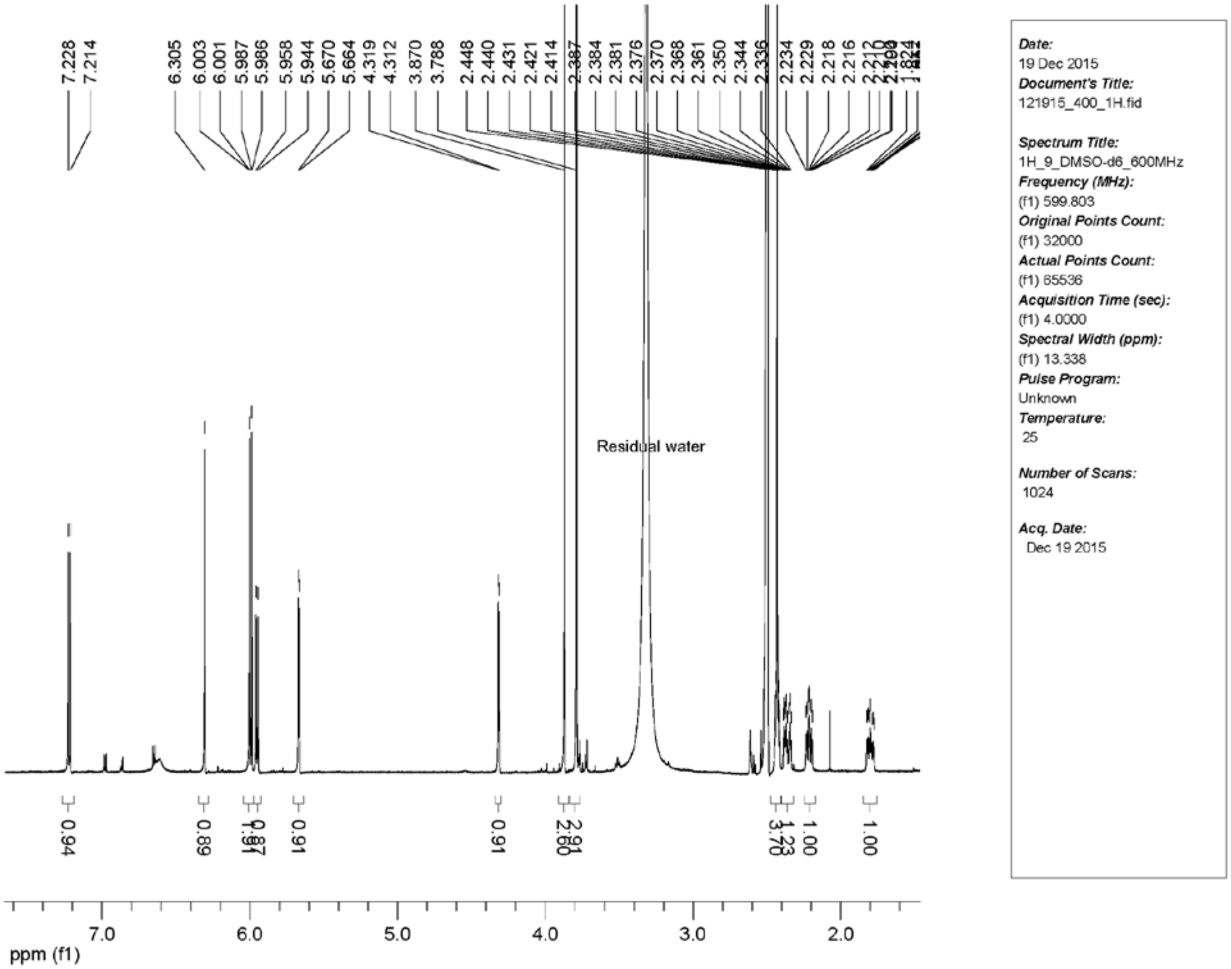

Residual water
ppm (f1)
7.0
6.0
5.0
4.0
3.0
2.0
0.94
0.89
0.91
0.91
1.00
1.00
Date:
19 Dec 2015
Document's Title:
121915_400_1H.fid
Spectrum Title:
1H_9_DMSO-d6_600MHz
Frequency (MHz):
(f1) 599.803
Original Points Count:
(f1) 32000
Actual Points Count:
(f1) 65536
Acquisition Time (sec):
(f1) 4.0000
Spectral Width (ppm):
(f1) 13.338
Pulse Program:
Unknown
Temperature:
25
Number of Scans:
1024
Acq. Date:
Dec 19 2015

b

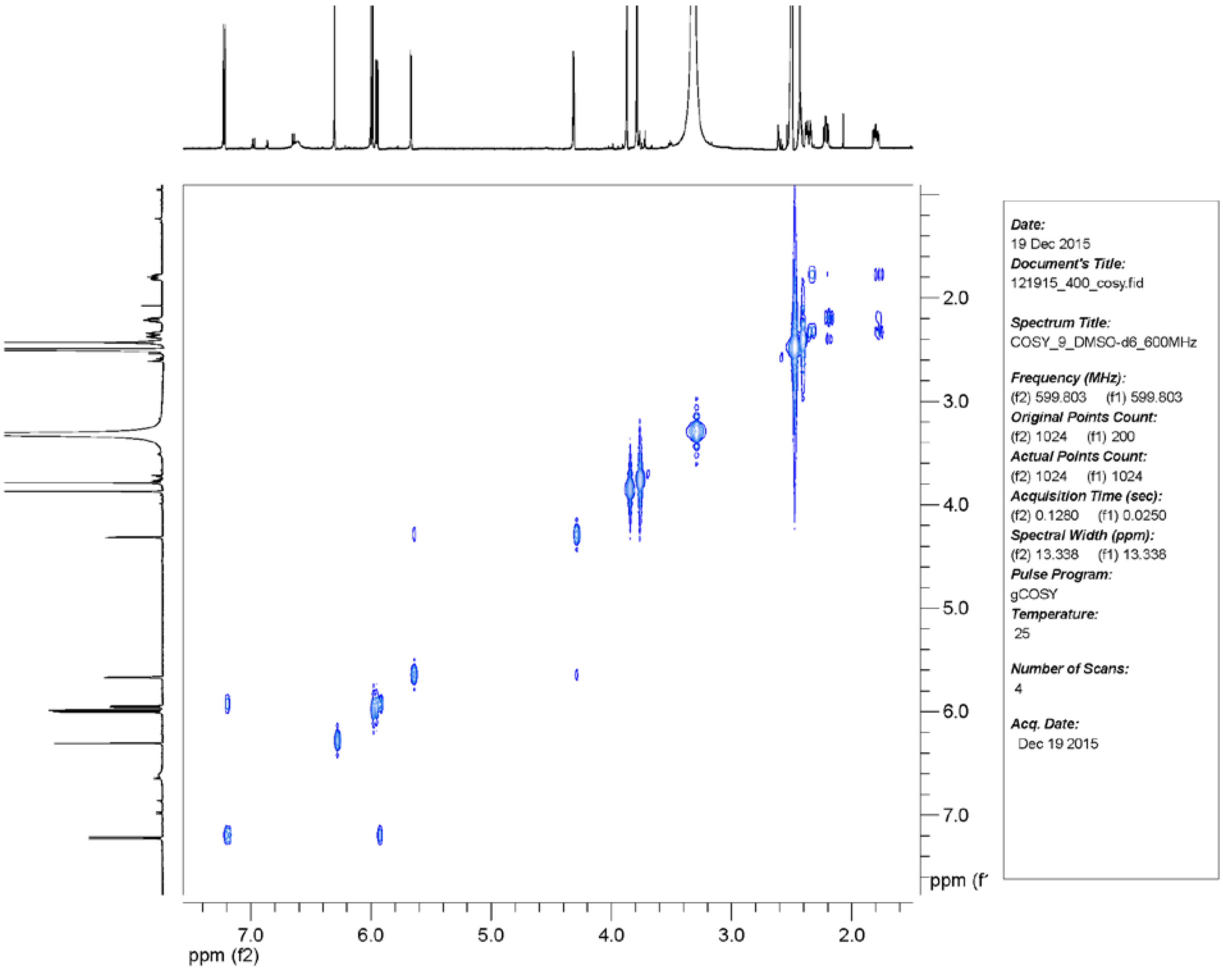

7.0
6.0
5.0
4.0
3.0
2.0
ppm (f2)
ppm (f1)
Date:
19 Dec 2015
Document's Title:
121915_400_cosy.fid
Spectrum Title:
COSY_9_DMSO-d6_600MHz
Frequency (MHz):
(f2) 599.803 (f1) 599.803
Original Points Count:
(f2) 1024 (f1) 200
Actual Points Count:
(f2) 1024 (f1) 1024
Acquisition Time (sec):
(f2) 0.1280 (f1) 0.0250
Spectral Width (ppm):
(f2) 13.338 (f1) 13.338
Pulse Program:
gCOSY
Temperature:
25
Number of Scans:
4
Acq. Date:
Dec 19 2015

c

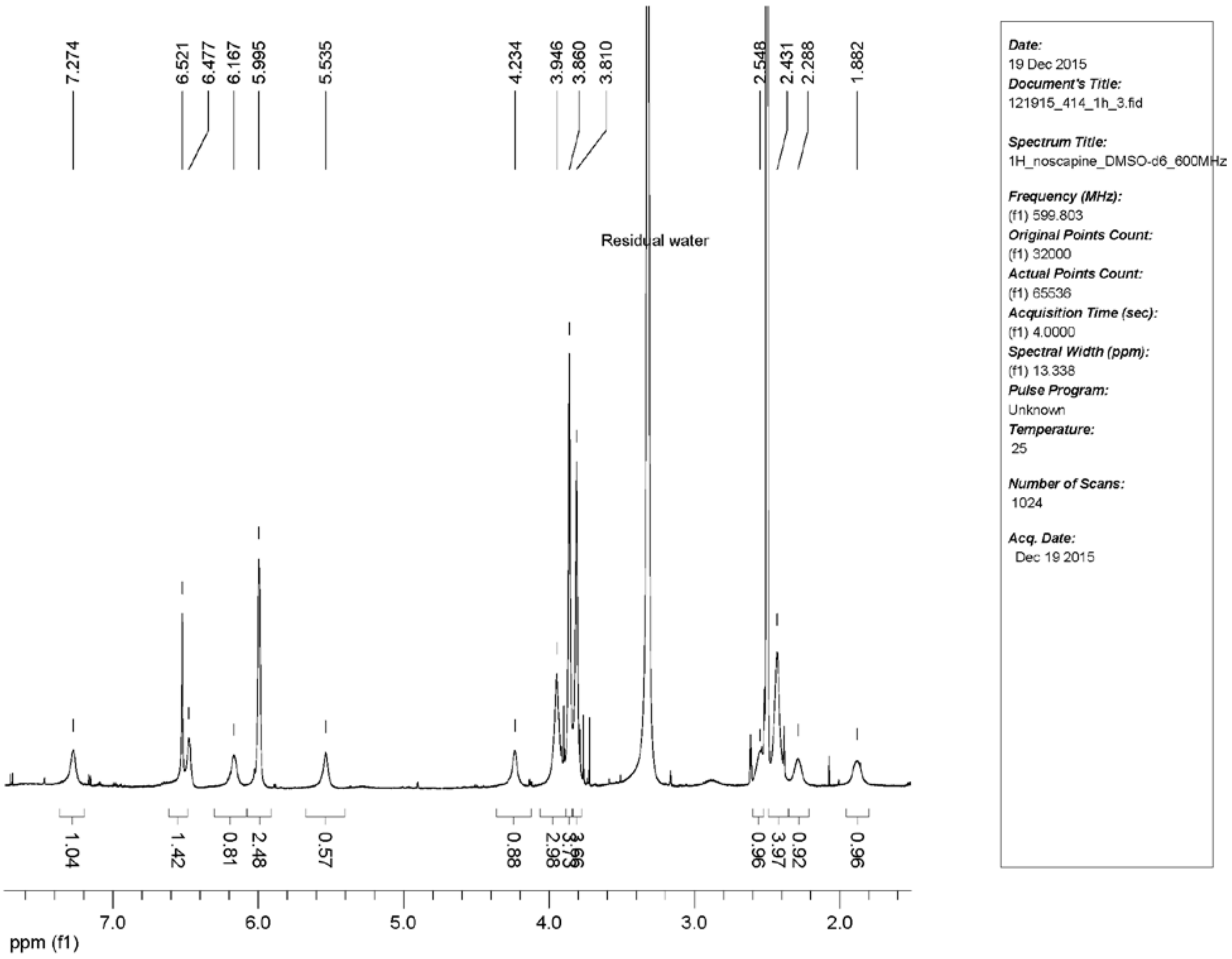

7.274
6.521
6.477
6.167
5.995
5.535
4.234
3.946
3.860
3.810
2.548
2.431
2.288
1.882
Residual water
1.04
1.42
0.81
2.48
0.57
0.88
2.98
0.96
3.97
0.92
0.96
7.0
6.0
5.0
4.0
3.0
2.0
ppm (f1)
Date:
19 Dec 2015
Document's Title:
121915_414_1h_3.fid
Spectrum Title:
1H_noscapine_DMSO-d6_600MHz
Frequency (MHz):
(f1) 599.803
Original Points Count:
(f1) 32000
Actual Points Count:
(f1) 65536
Acquisition Time (sec):
(f1) 4.0000
Spectral Width (ppm):
(f1) 13.338
Pulse Program:
Unknown
Temperature:
25
Number of Scans:
1024
Acq. Date:
Dec 19 2015

d

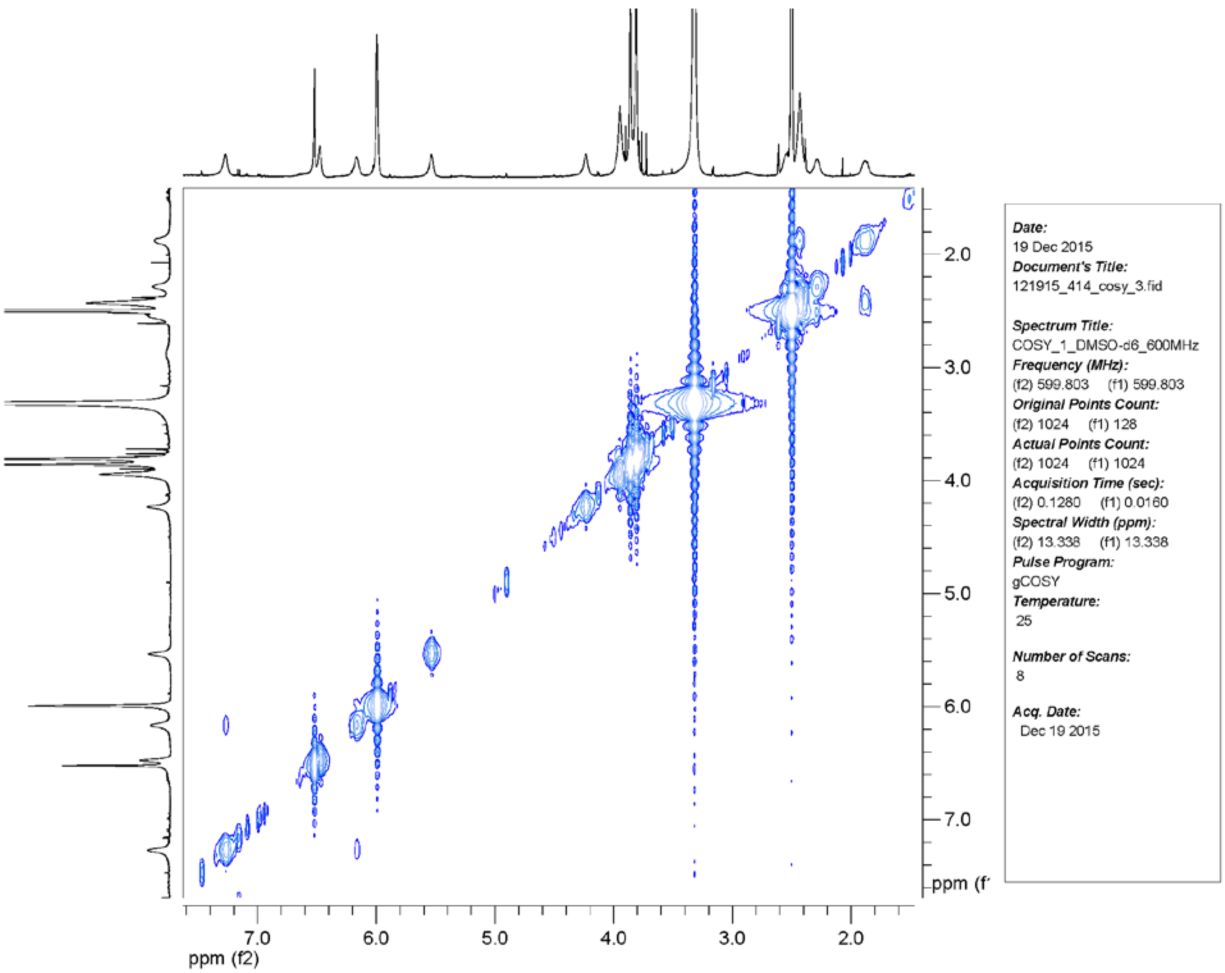

Supplementary Figure 9. NMR Spectra Data of Narcotoline, 9 & Noscapine, 1

(a) ^{1}H and (**b**) COSY NMR spectrum of **9**; **(c)** ^{1}H and (**d**) COSY NMR spectrum of **1**. ^{1}H and COSY NMR spectra were obtained at 600 MHz and recorded in DMSO-d6.

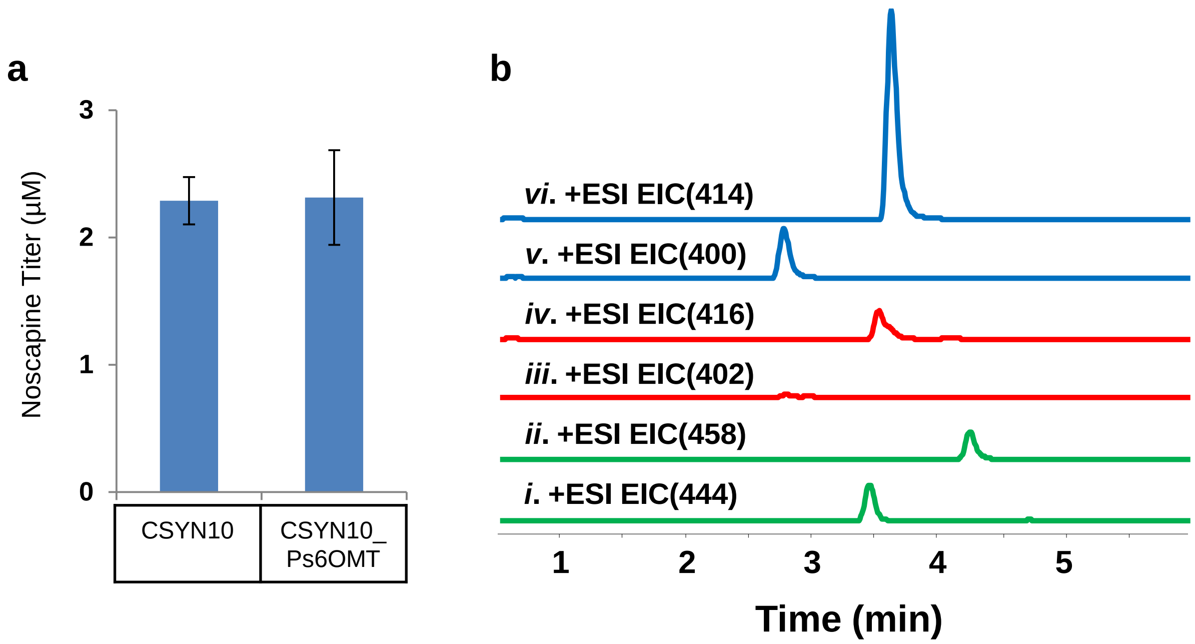

Supplementary Figure 10. LC-MS analysis of yeast strains engineered for the synthesis of 1 from 2

(a) Noscapine titer analyzed from engineered strains harboring different sets of expression cassettes. CSYN10: AtATR1, PsTNMT, CYP82X2, PsAT1 expressed from the chromosome; CYP82Y1, PsMT2, Ps6OMT, CYP82X1, PsCXE1, PsSDR1 expressed from a low-copy plasmid (Supplementary Data 1). CSYN10_Ps6OMT: AtATR1, PsTNMT, CYP82X2, PsAT1 expressed from the chromosome; CYP82Y1, PsMT2, Ps6OMT, CYP82X1, PsCXE1, PsSDR1 expressed from a low-copy plasmid (Supplementary Data 1). **(b)** EIC of m/z^+= (*i*) 444, (*ii*) 458, (*iii*) 402, (*iv*) 416, (*v*) 400, and (*vi*) 414 of **1**-producing yeast strain. All EIC traces are in the same scale. For all assays, yeast strains were fed 250 µM racemic **2** and grown in defined medium for 72 hours. Metabolites in the culture medium were analyzed by LC-MS/MS. The identities of metabolites were confirmed by comparison to the MS2 spectra of reported standards or by NMR spectroscopy analysis. All traces are representative of at least 3 biological replicates for each engineered yeast train.

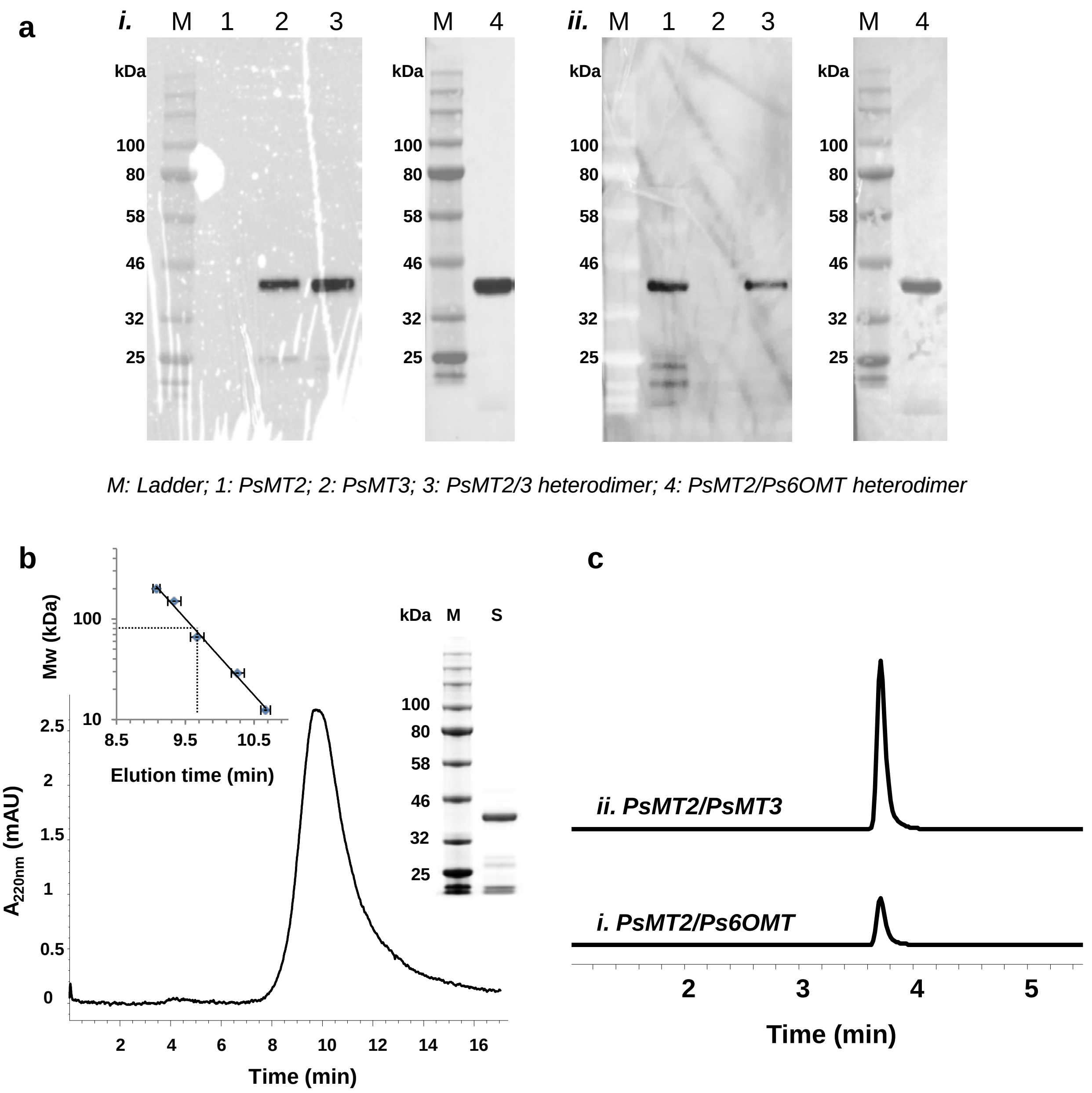

Supplementary Figure 11. Characterization of PsMT2/PsMT3 and PsMT2/Ps6OMT heterodimers

(a) Uncropped western blot analysis of the purified and concentrated 1) PsMT2 homodimer, 2) PsMT3 homodimer, 3) PsMT2/PsMT3 heterodimer, 4) PsMT2/Ps6OMT heterodimer with i) Anti-T7 tag® antibody and ii) Anti-6X His tag® antibody. Gels and blots are representative of two biological replicates. **(b)** Size-exclusion chromatography (SEC) analyses of the PsMT2/Ps6OMT heterodimer. The left inset is the calibration curve of the protein standards, with the retention time on the x-axis, and the molecular weight in log scale on the y-axis. The right insert is the SDS-PAGE analysis of M) ladder, and S) the purified and concentrated PsMT2/Ps6OMT heterodimer. The molecular weight of the His-tagged PsMT2 is 40.09 kDa, the T7-tagged PsMT3 is 38.70 kDa, and the T7-tagged Ps6OMT is 39.60 kDa. The calculated

molecular weight of the PsMT2/Ps6OMT complex is ~80 kDa, calculated based on at least three replicates. The error bars represent the standard deviation of the replicates. **(c)** EIC MRM using noscapine's highest characteristic precursor ion/product ion transition (414→220) of *in vitro* assays containing (i) PsMT2/Ps6OMT heterodimer, (ii) PsMT2/PsMT3 heterodimer. All traces are in the same scale and are representative of 3 biological replicates for each enzyme.

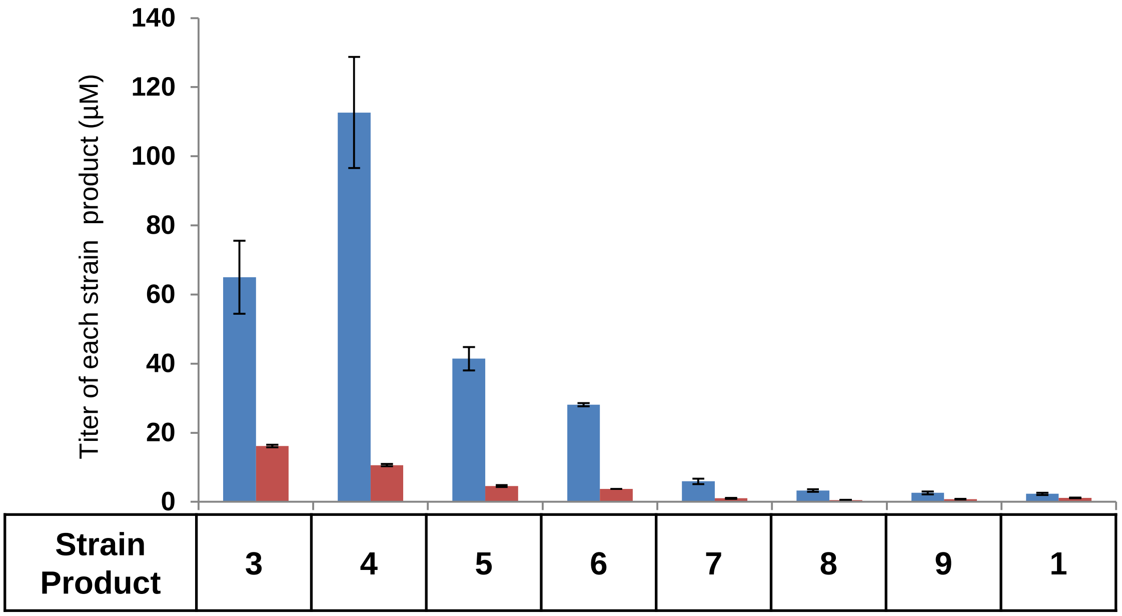

Supplementary Figure 12. Estimation of the conversion of (*S*)- and (*R*)-canadine by strains engineered to product major noscapine pathway metabolites

Titer of the final product in the **3**, **4**, **5**, **6**, **7**, **8**, **9**, and **1**-producing yeast strains (Supplementary Data 1). Blue indicates the titer of the corresponding metabolites when strains are fed (*S*)-canadine, and red indicates the titer of the corresponding metabolites when strains are fed with (*R*)-canadine. For all assays, yeast strains were fed 125 µM (*R*)- or (*S*)-enantiomer of **2** and grown in defined medium for 72 hours at 25°C. Metabolites in the culture medium were analyzed and quantified by LC-MS/MS. Bars represent mean values ± 1 s.d. of three biological replicates, and the error bars represent the standard deviation of the replicates.

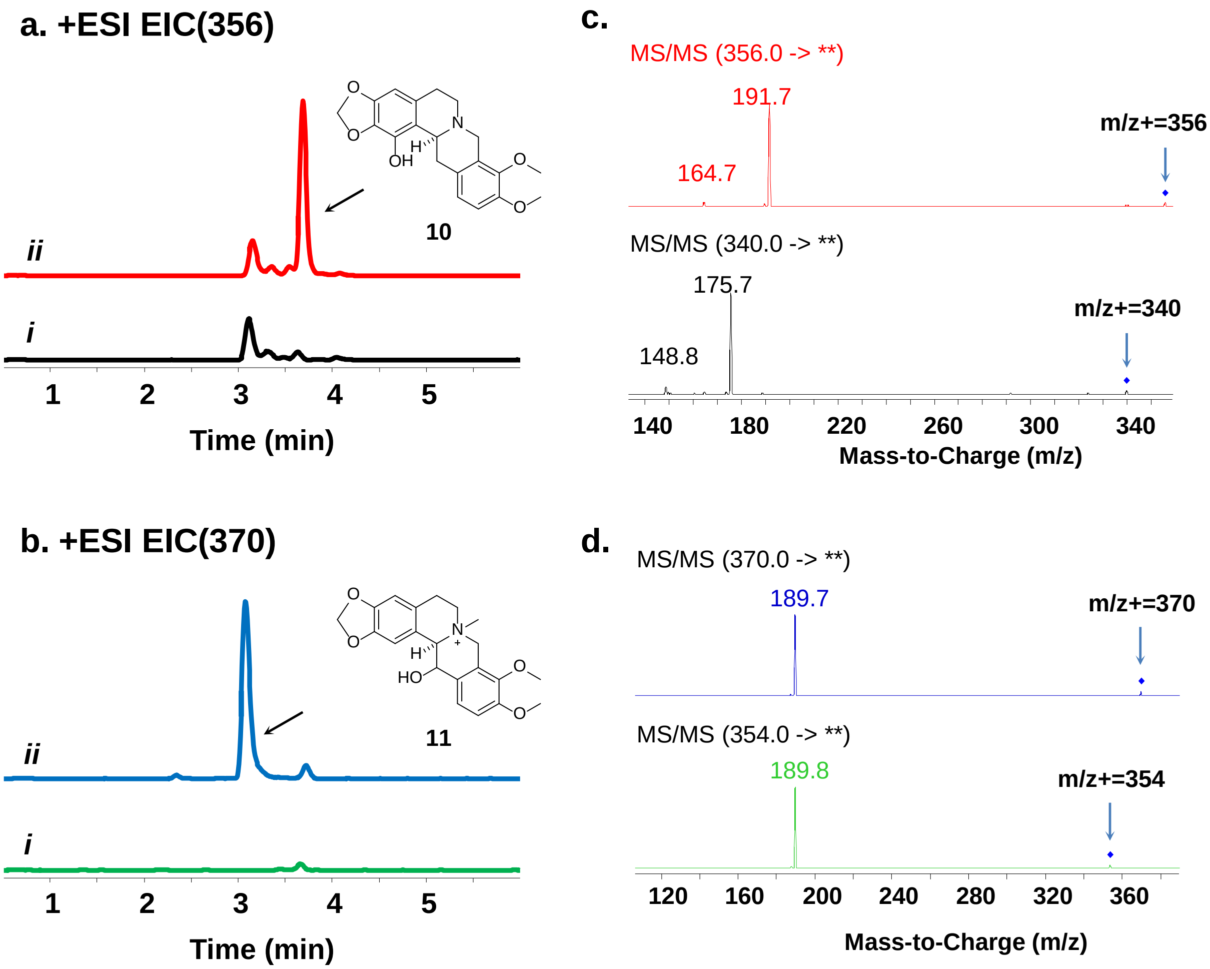

Supplementary Figure 13. LC-MS analysis of yeast strains engineered for the synthesis of 10 and 11

(a) EIC of m/z$^+$= 356 of (*i*) wild type yeast strain and (*ii*) strain expressing AtATR1 and CYP82Y1. **(b)** EIC of m/z$^+$=370 of (*i*) **3**-producing strain, (*ii*) strain expressing PsTNMT, AtATR1, and CYP82X2. **(c)** MS/MS spectrum of **2** (m/z$^+$=340, black) and **10** (m/z$^+$=356, red). **(d)** MS/MS spectrum of **3** (m/z$^+$=354, green) and **11** (m/z$^+$=370, blue). For all assays, yeast strains were fed 250 µM racemic **2** and grown in defined medium for 72 hours. Metabolites in the culture medium were analyzed by LC-MS/MS. All traces are representative of at least 3 biological replicates for each engineered yeast strain.

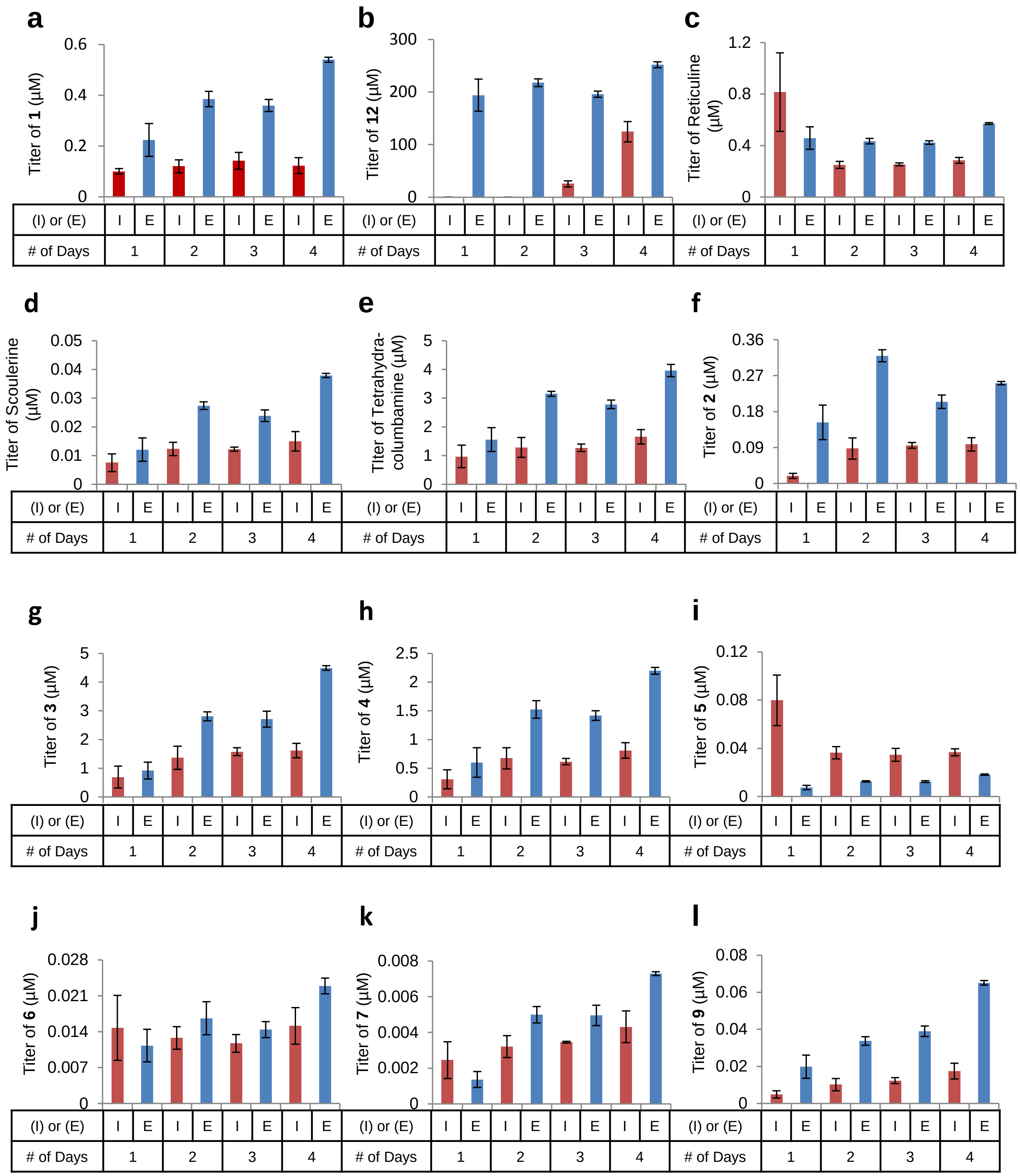

Supplementary Figure 14. Comparison of intracellular and extracellular concentrations of noscapine pathway metabolites

Titer of **(a) 1**, **(b) 12**, **(c)** reticuline, **(d)** scoulerine, **(e)** tetrahydracolumbamine, **(f) 2**, **(g) 3**, **(h) 4**, **(i) 5**, **(j) 6**, **(k) 7**, **(l) 9** in the cell pellets (intracellular, I, red) or in the medium (extracellular, E, blue) of CSYN16 cultured 1-4 days after induction. All traces are representative of 3 biological replicates for each sample, and the error bars represent the standard deviation of the replicates.

Supplementary Table 1. NMR Spectra Data of N-methylcanadine, 3 & 1-hydroxy-N-methylcanadine, 4

^{1}H and COSY NMR spectra were obtained at 600 MHz and recorded in CD_3CN. The numbering of **3** and **4** follows that of canadine as shown here.

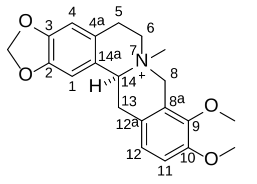

N-methylcanadine, 3

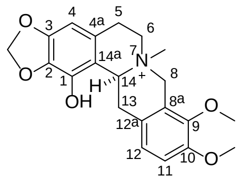

1-Hydroxy-N-methylcanadine, 4

	3			4		
No.	^{1}H δ [ppm]<br>(m, area, J_{HH} (Hz))	COSY	Reported ^{1}H δ [ppm]<br>(m, area, J_{HH} (Hz))<br>in CD_3OD[9**]	^{1}H δ [ppm]<br>(m, area, J_{HH} (Hz))	COSY	Reported ^{1}H δ [ppm]<br>(m, area, J_{HH} (Hz))<br>in CD_3OD[3]
1	6.79 (s, 1H)	-	6.83 (s, 1H)	-	-	-
2	-	-	-	-	-	-
-OCH_2O-	5.98 (d, 1H, 1.2)<br>6.00 (d, 1H, 1.2)	-	5.98 (s, 1H)<br>5.99 (s, 1H)	5.85(s, 2H)	-	5.91 (s, 1H)<br>5.92 (s, 1H)
3	-	-	-	-	-	-
4	6.75 (s, 1H)	-	6.80 (s, 1H)	6.28 (s, 1H)	-	6.31 (s, 1H)
4a	-	-	-	-	-	-
5	3.21 (m, 2H)	6-H	3.16 (m, 2H)*	3.06 (m, 1H)<br>3.19 (m, 1H)	6-H	3.16 (m, 2H)*
6	3.46 (m, 1H)<br>3.68 (m, 1H)	5-H	3.80 (m, 1H)<br>3.83 (m, 1H)	3.31 (m, 1H)<br>3.60 (m, 1H)	5-H	3.75 (m, 1H)<br>3.77 (m, 1H)
N-CH_3	3.16 (s, 3H)	-	3.27 (s, 3H)	3.14 (s, 3H)	-	3.24 (s, 3H)
8	4.60 (d, 1H, 16.2)<br>4.73 (d, 1H, 16.8)	-	4.84, 4.85 (broad, 2H)	4.67 (d, 1H, 16.2)<br>4.78 (d, 1H, 16.2)	-	4.84, 4.85 (broad, 2H)
8a	-	-	-	-	-	-
9	-	-	-	-	-	-
9-O-CH_3	3.85 (s, 3H)	-	3.89 (s, 3H)	3.85 (s, 3H)	-	3.89 (s, 3H)
10	-	-	-	-	-	-
10-O-CH_3	3.83 (s, 3H)	-	3.86 (s, 3H)	3.83 (s, 3H)	-	3.87 (s, 3H)
11	6.95 (d, 1H, 8.4)	12-H	6.97 (d, 1H, 8.4)	6.91 (d, 1H, 8.4)	12-H	6.95 (d, 1H, 8.4)
12	7.05 (d, 1H, 8.4)	11-H	7.07 (d, 1H, 8.4)	7.03 (d, 1H, 8.4)	11-H	7.06 (d, 1H, 8.4)
12a	-	-	-	-	-	-
13	3.08 (m, 1H)<br>3.42 (m, 1H)	14-H	3.19(m, 1H)<br>3.43 (m, 1H)	2.86 (dd, 1H, 11.4, 18)<br>3.49 (dd, 1H, 5.4, 18)	14-H	3.19 (m, 1H)<br>3.42 (m, 1H)
14	4.56 (m, 1H)	13-H	4.72 (m, 1H)	5.22 (m, 1H)	13-H	4.99 (m, 1H)
14a	-	-	-	-	-	-

*The assignment of chemical shifts of 5-H is missing[3]. The chemical shift of 5-H of **3** in DMSO-d6 is 3.16 (m, 2H)[4].

Supplementary Table 2. Two-way ANOVA of the effects of temperature and N-terminus engineering to the activity of CYP82Y1

Table Analyzed	Two-way ANOVA , not RM				
**Two-way ANOVA**	Ordinary				
**Alpha**	0.05				
**Source of Variation**	**% of total variation**	**P value**	**P value summary**	**Significant?**	
**Interaction**	0.01899	0.9381	ns	No	
**N-terminus Engineering**	16.95	0.0436	*	Yes	
**Temperature**	59.36	0.0021	**	Yes	
**ANOVA table**	**SS**	**DF**	**MS**	**F (DFn, DFd)**	**P value**
**Interaction**	0.09046	1	0.09046	F (1, 8) = 0.006421	P = 0.9381
**N-terminus Engineering**	80.74	1	80.74	F (1, 8) = 5.731	P = 0.0436
**Temperature**	282.7	1	282.7	F (1, 8) = 20.07	P = 0.0021
**Residual**	112.7	8	14.09		
**Number of missing values**	0				

Supplementary Table 3. NMR Spectra Data of 4'-O-Desmethylmacrantaldehyde, 5’

^{1}H, HSQC, and HMBC NMR spectra were obtained at 800 MHz, COSY spectrum was obtained at 600 MHz and recorded in CD_3CN. The numbering of **5’** follows that of noscapine as shown here.

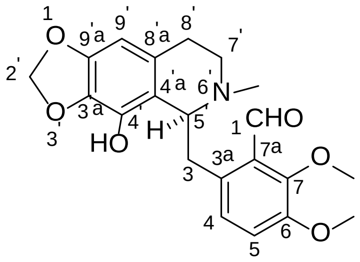

4'-O-Desmethylmacrantaldehyde, 5'

No.	^{1}H δ [ppm] (m, area, J_{HH} (Hz))	^{13}C δ [ppm]	COSY	^{1}H-^{13}C HMBC
1	10.22 (s br, 1H)	-	-	-
3	2.90 (dd, 1H, 9.6, 13.6)<br>3.20 (dd, 1H, 11.2, 14.4)	37.2	5'-H	C4, C4'a, C5'
3a	-	133.7	-	-
4	6.93(d, 1H, 8.8)	128.6	4-H	C3, C7a$_2$, C5, C6
5	7.14 (d, 1H, 8.8)	118.0	5-H	C3a, C7
6	-	152.0	-	-
6-O-CH_3	3.87 (s, 3H)	56.1	-	C6
7	-	152.7	-	-
7-O-CH_3	3.90 (s, 3H)	62.0	-	C7
7a	-	129.7	-	-
2'	5.88 (dd, 2H, 0.8, 4)	102.1	-	C3'a, C9'a
3'a	-	133.5	-	-
4'	-	-	-	-
4'a	-	120.1	-	-
5'	3.83 (m, 1H)	59.4	3-H	-
N-CH_3	2.19 (s, 3H)	43.5	-	C5', C7'
7'	2.62 (d, 1H, 11.2)<br>3.22 (m, 1H, 2.4, 4.8, 10.4)	45.6	8'-H	-
8'	2.34 (d, 1H, 16)<br>2.76 (m, 1H, 4, 6.4)	23.9	7'-H	C4'a, c8'a
8'a	-	129.3	-	-
9'	6.23 (s, 1H)	101.6	-	C3'a, C4'a, C9'a
9'a	-	148.5	-	-

Supplementary Table 4. NMR Spectra Data of 1,13-Dihydroxy-N-methylcanadine, 5

^{1}H, COSY, and HSQC NMR spectra were obtained at 600 MHz, HMBC NMR spectrum was obtained at 800 MHz and recorded in $CDCl_3$. The numbering of **5** follows that of canadine as shown here.

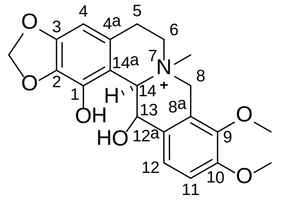

1,13-Dihydroxy-N-methylcanadine, 5

No.	^{1}H δ [ppm] (m, area, J_{HH} (Hz))	^{13}C δ [ppm]	COSY	^{1}H-^{13}C HMBC	Reported ^{1}H δ [ppm] (m, area, J_{HH} (Hz)) in 4:1 $CDCl_3$+CD_3CN[2]	Reported ^{13}C δ [ppm] in 4:1 $CDCl_3$+CD_3CN[2]
1	-	-	-	-	-	-
2	-	135.3	-	-	-	135.1
-O-CH_2-O-	5.86 (d, 2H, 4.8)	100.8	-	C2, C3	5.80 (d, 1H, 1.5)<br>5.84(d, 1H, 1.5)	99.8
3	-	149.3	-	-	-	148.5
4	6.05 (s, 1H)	97.1	-	C2, C3, C5, C14, C14a	5.85 (s, 1H)	92.4
4a	-	120.9	-	-	-	119.1
5	2.92 (dd, 1H, 4.8, 24)<br>3.15 (m, 1H)	23.8	6-H	C4, C4a, C6, C14a	2.95 (dd, 1H, 6.2, 17.1)<br>3.15 (m, 1H, 295, 3,42)	24.1
6	3.34 (m, 2H)	53.1	5-H	-	3.14 (m, 1H, 2.95, 3.42)<br>3.42 (td, 1H, 2.95, 3.16)	53.6
N-CH_3	3.38 (s, 3H)	51.5	-	C6, C8, C14	3.26 (s, 3H)	51.1
8	4.93 (s, 2H)	60.4	-	C8a, C12a, C14	4.73 (d, 1H, 15.3)<br>4.82 (d, 1H, 15.3)	61.0
8a	-	120.3	-	-	-	118.8
9	-	145.1	-	-	-	144.9
9-O-CH_3	3.874 (s, 3H)	61.4	-	C9	3.876 (s, 3H)	61.2
10	-	152.4	-	-	-	152.1
10-O-CH_3	3.865 (s, 3H)	55.6	-	C10	3.89 (s, 3H)	56.07
11	6.95 (d, 1H, 8.4)	113.2	12-H	C9, C10, C12a	7.03 (d, 1H, 8.5)	114.1
12	7.20 (d, 1H, 8.4)	124.5	11-H	C8a, C10, C13	7.26 (d, 1H, 8.5)	124.9
12a	-	126.6	-	-	-	127.4
13	4.88 (d, 1H, 5.4)	72.8	14-H	C8a, C12a, C14a	4.92 (d, 1H, 6.5)	73.8
14	5.28 (d, 1H, 4.8)	69.3	13-H	C13, C14a	5.02 (d, 1H, 5.8)	70.3
14a	-	115.3	-	-	-	117.3

Supplementary Table 5. NMR Spectra Data of 1-hydroxy-13-O-acetyl-N-methylcanadine, 6

^{1}H, COSY, HSQC, and HMBC NMR spectra were obtained at 600 MHz and recorded in CD_3CN.

6 is in formate form, and the numbering of **6** follows that of canadine as shown here.

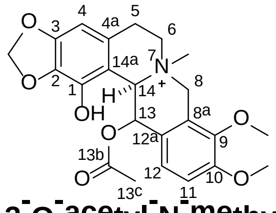

1-Hydroxy-13-O-acetyl-N-methylcanadine, 6

No.	^{1}H δ [ppm] (m, area, J_{HH} (Hz))	^{13}C δ [ppm]	COSY	^{1}H-^{13}C HMBC	Reported ^{1}H δ [ppm] (m, area, J_{HH} (Hz)) in 4:1 $CDCl_3$+CD_3CN[2]	Reported ^{13}C δ [ppm] in 4:1 $CDCl_3$+CD_3CN[2]
1	-	-	-	-	-	139.2
2	-	135.5	-	-	-	135.5
-O-CH_2-O-	5.84 (d, 2H, 6.6)	102.2	-	C2, C3	5.97 (s, 2H)	102
3	-	150.1	-	-	-	149.9
4	6.21 (s, 1H)	99.7	-	C2, C3, C5, C14a	6.32 (s, 1H)	101.5
4a	-	123.9	-	-	-	122.2
5	3.05 (m, 2H)	24.0	6-H	-	3.09 (m, 1H)<br>3.15 (m, 1H)	23.5
6	3.26 (m, 1H)<br>3.53 (m, 1H)	54.9	5-H	-	3.30 (m, 1H)<br>3.37 (m, 1H)	53.2
N-CH_3	3.21 (s, 3H)	52.8	-	C6, C8, C14	3.37 (s, 3H)	51.8
8	4.86 (s, 2H)	61.0	-	C6, C8a, C9, C12, C14	4.92 (d, 1H, 15.3)<br>4.96 (d, 1H, 15.3)	60.6
8a	-	121.5	-	-	-	120.8
9	-	145.8	-	-	-	145.6
9-O-CH_3	3.86 (s, 3H)	61.6	-	C9	3.90 (s, 3H)	61.2
10	-	153.8	-	-	-	153.3
10-O-CH_3	3.87 (s, 3H)	56.5	-	C10	3.89 (s, 3H)	56.1
11	7.08 (d, 1H, 8,4)	114.5	12-H	C9, C10, C12a	7.03 (d, 1H, 8.6)	113.9
12	7.03 (d, 1H, 8.4)	125.5	11-H	C10, C8a, C13	7.01 (d, 1H, 8.6)	124.6
12a	-	124.7	-	-	-	122.8
13	6.13 (d, 1H, 6.6)	72.2	14-H	C8a, C11, C12, C12a, C13b, C14a	6.10 (d, 1H, 6.8)	71.4
13b	-	170.7	-	-	-	170.2
13c	2.03 (s, 3H)	21.2	-	C13b	2.10 (s, 3H)	20.9
14	5.48 (d, 1H, 6.6)	65.7	13-H	C4a,C6, C12a, C13, C14a	5.59 (d, 1H, 6.8)	64.8
14a	-	114.5	-	-	-	113

Supplementary Table 6. NMR Spectra Data of 4'-O-Desmethyl-3-O-Acetylpapaveroxine, 7

^{1}H and 2D NMR spectra were obtained at 600 MHz, ^{13}C NMR spectrum was obtained at 500 MHz and recorded in CD_3CN. The numbering of **7** follows that of noscapine as shown here.

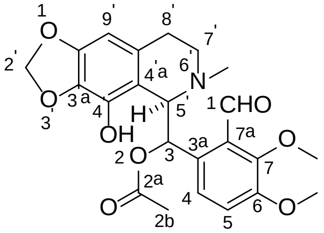

4'-O-Desmethyl-3-O-acetylpapaveroxine, 7

No.	^{1}H δ [ppm] (m, area, J_{HH} (Hz))	^{13}C δ [ppm]	COSY	^{1}H-^{13}C HMBC
1	10.06 (s, 1H)	191.74	-	C7a
2a	-	170.73	-	-
2b	1.99 (s, 3H)	21.30	-	C2a
3	6.36 (d, 1H, 5.4)	76.09	5'-H	C2a, C3a, C4, C4'a, C5', C7a
3a	-	131.04	-	-
4	7.09 (s, 2H)	125.42	-	C3, C3a, C6, C7, C7a
5		116.15		
6	-	153.22	-	-
6-O-CH_3	3.85 (s, 3H)	56.49	-	C6
7	-	150.98	-	-
7-O-CH_3	3.82 (s, 3H)	62.22	-	C7
7a	-	131.38	-	-
2'	5.85 (d, 1H, 1.2)<br>5.88 (d, 1H, 1.2)	102.04	-	C3'a, C9'a
3'a	-	133.67	-	-
4'	-	138.77	-	-
4'a	-	116.86	-	-
5'	4.14 (d, 1H, 5.4)	62.27	3-H	C3, C3a, C4', C4'a, N-CH_3, C7', C8'a
N-CH_3	2.37 (s, 3H)	45.76	-	C5', C7'
7'	2.90 (td, 1H, 4.2, 10.2)<br>2.21 (dt, 1H, 3.6, 10.8)	51.45	8'-H	N-CH_3, C8', C5', C8'a
8'	2.05 (ddd, 1H, 4.2, 10.8)<br>2.32 (dt, 1H, 3.6, 15.6)	28.95	7'-H	C7', C9', C4'a, C8'a
8'a	-	133.11	-	-
9'	6.17 (s, 1H)	101.11	8'-H	C3'a, C4', C4'a, C8', C9'a
9'a	-	148.03	-	-

Supplementary Table 7. NMR Spectra Data of Narcotoline, 9 & Noscapine, 1

^{1}H and COSY NMR spectra were obtained at 600 MHz and recorded in DMSO-d6. The numbering of **9** and **1** follows that of noscapine as shown here.

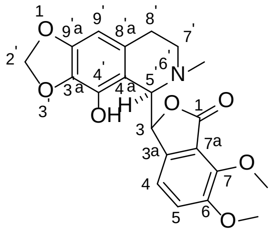

Narcotoline, 9

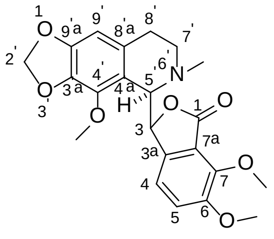

Noscapine, 1

No.	**9** ^{1}H δ [ppm] (m, area, J_{HH} (Hz))	**9** COSY	**1** ^{1}H δ [ppm] (area)*	**1** COSY	**1** Reported ^{1}H δ [ppm] (m, area, J_{HH} (Hz)) in $CDCl_3$[5,6]
1	-	-	-	-	-
3	5.67 (dd, 1H, 0.6, 9.6)	H-5'	5.54 (1H)	H-5'	5.57 (d, 1H, 4.5)
3a	-	-	-	-	-
4	5.95 (dd, 1H, 0.6, 8.4)	H-5	6.17 (1H)	H-5	6.09 (d, 1H, 3.8)
5	7.22 (d, 1H, 8.4)	H-4	7.27 (1H)	H-4	6.97 (d, 1H, 5.5)
6	-	-	-	-	-
6-O-CH_3	3.79 (s, 3H)	-	3.81 (3H)	-	3.87 (s, 3H)
7	-	-	-	-	-
7-O-CH_3	3.87 (s, 3H)	-	3.86 (3H)	-	4.10 (s, 3H)
7a	-	-	-	-	-
2'	5.99 (dd, 2H, 1.2, 9.6)	-	6.00 (2H)	-	5.94 (d, 2H, 0.8)
3'a	-	-	-	-	-
4'	-	-	-	-	-
4'-O-CH_3	-	-	3.95 (3H)	-	4.06 (s, 3H)
4'a	-	-	-	-	-
5'	4.31 (d, 1H, 4.2)	H-3	4.23 (1H)	H-3	4.40 (d, 1H, 3.7)
N-CH_3	2.43 (s, 3H)	-	2.43 (s, 3H)	-	2.56 (s, 3H)
7'	2.36 (m, 1H)<br>2.43 (m, 1H)	H-8'	2.29 (1H)<br>2.55 (1H)	H-8'	2.6 (m, 1H)<br>2.3 (m, 2H)
8'	1.80 (m, 1H)<br>2.21 (m, 1H)	H-7'	1.88 (1H)<br>2.43 (1H)	H-7'	1.9 (m, 1H)
8'a	-	-	-	-	-
9'	6.31 (s, 1H)	-	6.52 (1H)	-	6.31 (s, 1H)
9'a	-	-	-	-	-

* All the signals of **1** appear to be broad peaks in DMSO-d6, possibly due to chemical exchange.

Supplementary Table 8. MRM transitions used to quantify alkaloids in LC-MS/MS analysis

Compound	Quantifier MRM Transition			Qualifier MRM Transition		
	Precursor → Product Ion	Fragmentor	Collision Energy	Precursor → Product Ion	Fragmentor	Collision Energy
Reticuline	330→192	120	19	330→137	120	31
Scoulerine	328→151	135	30	328→178	135	29
Tetrahydrocolumbamine	342→178	135	29	342→163	135	29
**1**	414→220	138	23	414→353	138	23
**2**	340→176	135	29	340→149	135	25
**3**	354→190	135	25	354→188	135	25
**4**	370→206	135	25	-*	-*	-*
**5**	386→206	135	25	-*	-*	-*
**6**	428→206	135	25	428→368	135	25
**7**	444→206	138	23	444→398	138	23
**8**	402→206	138	23	-*	-*	-*
**9**	400→206	138	23	400→339	138	23
**12**	288→164	122	15	288→123	122	31

*The qualifier transitions of **4**, **5,** and **8** are not determined; and the identities of **4**, **5,** and **8** are confirmed by the retention time.

Supplementary References:

1 Winzer, T. *et al.* A Papaver somniferum 10-gene cluster for synthesis of the anticancer alkaloid noscapine. *Science* **336**, 1704-1708, (2012).

2 Dang, T.-T. T., Chen, X. & Facchini, P. J. Acetylation serves as a protective group in noscapine biosynthesis in opium poppy. *Nat. Chem. Biol.* **11**, 104-106, (2015).

3 Dang, T. T. & Facchini, P. J. CYP82Y1 is N-methylcanadine 1-hydroxylase, a key noscapine biosynthetic enzyme in opium poppy. *J. Biol. Chem.* **289**, 2013-2026, (2014).

4 Binutu, O. A. & Cordell, G. A. Constituents of zanthoxylum sprucei. *Pharm. Biol.* **38**, 210-213, (2000).

5 Bulduk, I. & Taktak, F. Isolation and characterization of antitumor alkaloid from poppy capsules (papaver somniferum). *J. Chem.* http://www.hindawi.com/journals/jchem/2013/493870, (2013).

6 Janssen, R. H. A. M. *et al.* Assignments of H-1 and C-13 NMR resonances of some isoquinoline alkaloids .2. *Phytochemistry* **29**, 3331-3339, (1990).

7 Hawkins, K. M. & Smolke, C. D. Production of benzylisoquinoline alkaloids in Saccharomyces cerevisiae. *Nat. Chem. Biol.* **4**, 564-573, (2008).

8 Alberti, S., Gitler, A. D. & Lindquist, S. A suite of Gateway cloning vectors for high-throughput genetic analysis in Saccharomyces cerevisiae. *Yeast* **24**, 913-919, (2007).

9 Thodey, K., Galanie, S. & Smolke, C. D. A microbial biomanufacturing platform for natural and semisynthetic opioids. *Nat. Chem. Biol.* **10**, 837-844, (2014).

10 Galanie, S. & Smolke, C. D. Optimization of yeast-based production of medicinal protoberberine alkaloids. *Microb. Cell Fact.* **14**, 144, (2015).
